# The Politics of Regulating Foods for Infants and Young Children: A Case Study on the Framing and Contestation of Codex Standard-Setting Processes on Breast-Milk Substitutes

**DOI:** 10.34172/ijhpm.2021.161

**Published:** 2021-11-20

**Authors:** Monique Boatwright, Mark Lawrence, Cherie Russell, Katheryn Russ, David McCoy, Phillip Baker

**Affiliations:** ^1^School of Exercise and Nutrition Sciences, Deakin University, Geelong, VIC, Australia.; ^2^Institute for Physical Activity and Nutrition, School of Exercise and Nutrition, Deakin University, Geelong, VIC, Australia.; ^3^University of California, Davis, CA, USA; ^4^Centre for Primary Care and Public Health, Queen Mary University, London, UK.

**Keywords:** Breast-Milk Substitutes, Codex, International Food Standards, Follow-Up Formula, Political Economy, Commercial Determinants of Health

## Abstract

**Background:** Breastfeeding is important for the health and development of the child, and for maternal health, in all country contexts. However, global sales of breast-milk substitutes (BMS), including infant, follow-up and toddler formulas, have ‘boomed’ in recent decades. This raises the importance of international food standards established by the Codex Alimentarius Commission (Codex) on the safety, composition and labelling of BMS. Such standards appear to be strongly contested by governments, industry and civil society groups, yet few studies have investigated the politics of Codex standard-setting processes. The aim of this paper is to understand who participates in decision-making, and how actors frame and contest proposals to revise the Codex Standard on Follow-up Formula (FUF).

**Methods:** We adopted a case study design involving two steps. First, we enumerated government, industry, civil society, and international organization stakeholders participating in standard-setting processes of the Codex Committee on Nutrition and Foods for Special Dietary Uses (CCNFSDU). Second, we conducted a framing analysis of stakeholder inputs during the FUF standard revision in CCNFSDU meetings. Publicly available online meeting reports (2015-2019) were retrieved, analyzed using a theoretical framework, and organized thematically.

**Results:** High-income country (HIC) delegates greatly outnumbered those from other country income categories. Industry representation was higher compared with other observer categories. Member state delegations included more industry representation than civil society representation, and were occasionally the only member state delegates. Industry stakeholders framed arguments in terms of trade implications, science, and flexible standards. Civil society groups used public health, science, and pro-breastfeeding frames.

**Conclusion:** Codex BMS standard-setting procedures are dominated by HICs and industry groups. Limited representation of civil society, and of low- and middle-income countries (LMICs), suggest actions are needed to substantially increase support for their involvement at Codex. Such representation may help to counteract power asymmetries and commercial influences on food standards for infants and young children.

## Background

**Key Messages**

** Implications for policy makers**

- This paper provides evidence that high-income countries (HICs), and the baby food industry, hold asymmetrical power within the Codex Alimentarius Commission (Codex) Committee on Nutrition and Foods for Special Dietary Uses (CCNFSDU), which sets international standards for infants and young child foods.
- There is a low ratio of health ministry relative to other ministry (agriculture, commerce, trade etc) representation in country delegations to the CCNFSDU; and a low ratio of civil society observers compared with industry observers represented in setting these standards.
- Substantial increases in financial and technical support are needed for greater representation of low- and middle-income countries (LMICs), and for stronger civil society participation in Codex; further actions are needed to build greater awareness of Codex standard-setting processes for foods for infants and young children among public health and consumer organizations, and to encourage their stronger engagement in these processes, to protect infant and young child nutrition.

** Implications for the public**

This research contributes to literature which advocates for greater public health representation and protections in the process of developing international food standards for infants and young children. It raises public awareness of strategies used by the formula industry to market breast-milk substitutes (BMS) and reiterates the importance of the International Code of Marketing of Breast-milk Substitutes (The Code) and subsequent World Health Assembly (WHA) resolutions to retain a strong foothold in Codex Alimentarius Commission (Codex) BMS guidelines and standards. The research also highlights the addition of sweeteners and inadequate protein levels as guided by Codex standards, and the health implications of cross-promotion by infant formula (IF) companies. In view of this, we call for an increased engagement by public-interest civil society groups to help protect breastfeeding and scrutinize industry influence on ultra-processed foods for infants and young children, and additional funding to support the involvement of developing countries in the Codex Committee on Nutrition and Foods for Special Dietary Uses (CCNFSDU) proceedings.

The World Health Organization (WHO) recommends infants begin breastfeeding within the first hour of life, are then exclusively breastfed for six months, followed by the introduction of safe and nutritionally adequate complementary foods while breastfeeding continues for up to 2 years of age and beyond.^1,2^ The WHO’s 2003 Global Strategy on Infant and Young Child Feeding encourages governments to promote, protect and support breastfeeding,^3^ including incorporating the International Code of Marketing of Breast-milk Substitutes (*The Code*) and subsequent relevant World Health Assembly (WHA) resolutions into national legislation.^3^

Breastfeeding reduces the child’s risk of diarrhoea, pneumonia and dental malocclusions, with growing evidence suggesting it may lower the risk of obesity and type-2 diabetes, and help children realise their full cognitive potential.^4^ Breastfeeding also reduces the mother’s risk of breast and ovarian cancers, type-2 diabetes, and increases birth spacing.^5^ In 2015, it was estimated that 823 000 annual deaths could have been avoided in 75 low-income (LICs) and low- and middle-income countries (LMICs) if breastfeeding was scaled-up to near universal levels.^4^ Not breastfeeding generates economic losses of US$341.3 billion annually, resulting from higher healthcare costs, premature mortality and lost productivity.^6^ Nevertheless, the world exclusive breastfeeding rate <6 months rose from 35% to 42% between 2005 and 2018,^7^ albeit too slowly to meet the WHA global nutrition target of 50% by 2025.^8^

In 2016, the WHO issued technical *Guidance on the Inappropriate Promotion of Foods for Infants and Young Children*, defining cross-promotion as a ‘form of marketing promotion where customers of one product … are targeted with promotion of a related product’^9^ (p. 30).This incorporates near-identical brand names and packaging and labelling designs, to cross-promote infant formula (IF), Follow-up Formula (FUF), toddler milks, complementary foods and other products across the entire branded product range.^10,11^ It is also ‘an effective strategy for companies to continue indirect promotion of IF where national legislation or regulations prohibit direct marketing of such products’^9^ (p. 10).The WHO technical guidance was “welcomed with appreciation”in Resolution WHA69.9, wording that met resistance from the United States, European Union (EU) and New Zealand (NZ), and industry groups who claimed it does not obligate governments to implement it as an update to *The Code*.^12-14^ Nonetheless, the resolution urges member states to implement the guidance and calls upon manufacturers and distributors to adhere to the guidance and end the inappropriate promotion of breast-milk substitutes (BMS).^15^

As established by Resolution WHA69.9, BMS covered by *The Code*, include any milks (and milk replacements, such as fortified soy milks), that are specifically marketed for feeding infants and young children up to 3 years of age, including infant, follow-up, toddler and specialised formulas, in either powdered or liquid form.^16-18^ Long-standing evidence demonstrates that the aggressive marketing, including promotion of BMS, undermines breastfeeding exclusivity and duration by using powerful techniques to influence healthcare professionals and caregivers.^19^ In some jurisdictions, milk formulas are portrayed as similar (even superior) to breastmilk, with many unsubstantiated claims of health and development benefits for the child.^9,18-23^ Because some women may choose not to breastfeed, or are unable to because of work commitments, other societal pressures,^19^ and a small number of medical reasons, IFs are required for infant nutrition.^24^ Subsequently, BMS are made available as highly regulated food products. Since the mid-1980s the marketing of follow-up and toddler milks has become much more intensive, with sales of these categories ‘booming’ worldwide.^25^ This is despite WHO having long maintained these products are unnecessary and unsuitable as replacements for continued breastfeeding.^26^ Furthermore, these are ultra-processed beverage products, often high in added sugars (excluding lactose).^23,27,28^

The Codex Alimentarius Commission (Codex) was established by the United Nation’s (UN’s) Food and Agriculture Organization (FAO) and the WHO and has a dual mandate to protect the safety and health of consumers while harmonizing standards to facilitate global trade.^29^ This includes the development of ‘commodity specific’ standards for infant and specialised formulas (CXS 72-1981)^16^ and FUF (CXS 156-1987)^17^ that stipulate product composition, safety, labelling and other requirements that affect infant and young child feeding practices and nutrition.^29,30^ In addition, the WHA “requests the FAO/WHO Codex Alimentarius Commission to give full consideration, within the framework of its operational mandate, to action it might take to improve the quality standards of infant foods, and to support and promote the implementation of the International Code”^31^ (p. 18).Codex also includes the development of non-specific ‘horizontal standards,’ which compliment commodity standards, such as the General Standard for the Labelling of Pre-packaged Foods (CXS 1-1985).^32^ Although Codex standards are voluntary for member states to implement, they are frequently used in trade and investment agreements which ultimately creates obligations for countries to base national food regulations on standards designed to facilitate trade.^33^ Consequently, many LICs and LMICs adopt Codex standards verbatim into national food standards and legislation.^33,34^

This raises the significance of terms and their definitions as used within Codex standards, for instance ‘label’ and ‘labelling,’ because of possible trade implications or conversely, commercial opportunities, such as cross-promotion.^10,33,35^ For example, ‘label’ refers to “any tag, brand, mark, pictorial or other descriptive matter, written, printed, stencilled, marked, embossed or impressed on, or attached to, a container of food”^35^ (p. 2). Whereas, ‘labelling’ encompasses the meaning of ‘label,’ but extends to “…matter that is present on the label, accompanies the food, or is displayed near the food, including that for the purpose of promoting its sale or disposal”^35^ (p. 2).

In 1995, the importance of Codex further increased with the establishment of the World Trade Organization’s (WTO’s) Agreements on Sanitary and Phytosanitary Measures and the Technical Barriers to Trade which reference and use Codex standards as benchmarks in trade negotiations, and disputes.^36-42^ Although Codex standards were originally considered a minimum standard or regulatory “floor,” WTO wording effectively makes them a maximum regulatory requirement, or “ceiling,” meaning that countries choosing to adopt higher standards may be challenged and threatened with the possibility of trade disputes and penalties.^33,42-44^ As Codex standard-setting processes became more important, it became more politicized, with governments, industry and civil society groups regularly contesting the development and revision of standards.^37,38^

Currently, there are 189 member states (188 countries and the EU) with voting rights, and 236 non-state actors with observer status and no voting rights, involved in Codex decision-making processes.^32,45^ Industry representatives (BMS companies, dairy industry, other food industries, associations representing manufacturers, industry-sponsored consumer organizations) are commonly embedded within member state delegations where they act as advisors, and hence influence decision-making and voting by member states.^33,46-51^ Other stakeholders, such as public-interest non-governmental organizations (NGOs), and academics, can be involved in the same capacity. Observers, including intergovernmental organizations (IGOs), industry groups, and NGOs, are without voting rights, but can influence decisions via verbal and written collaborations with member state delegations, and may be permitted by the Chairperson to address the Committee.^33,51^

The structure of Codex incorporates the Commission, Executive Committee and subsidiary bodies, such as the Codex Committee on Nutrition and Foods for Special Dietary Uses (CCNFSDU).^52^ There is an annual meeting of the CCNFSDU and electronic working groups (eWG), or physical working groups (pWG), who meet throughout the year to develop, consider, and make amendments to standards, and submit proposals to Codex.^45,51-53^ During standard-setting proceedings, Codex may call on independent expert advisory bodies, such as the Joint FAO/WHO Expert Meetings on Nutrition (JEMNU), to carry out food-related scientific research on behalf of the CCNFSDU.^38^ However, member states are also able to provide data, or facilitate research outside of these bodies, which can often reflect evidence submitted by industry groups.^38^ How Codex determines the level of competence and independence of “experts” outside of JEMNU, remains unclear.

Despite the importance of *The Code* referring to Codex to support its implementation, and to the protection of infant and young child nutrition, to date, few studies have investigated the politics of Codex standard-setting processes, with some exceptions.^33,40,46,47,54,55^ This is an important gap in the literature, given the impact Codex standards have on global infant and young child feeding practices, nutrition, and child and maternal health. Addressing this gap is also key to understanding how industries exercise power in ways that may undermine public health, known as the commercial determinants of health.^56^

 This study aims to understand who influences Codex standard-setting processes and how different stakeholders contest the draft revision of the Codex Standard for FUF for older infants (FUF-OI), aged 6-12 months; and FUF for young children (FUF-YC), aged 12-36 months. To achieve this, we take two steps. First, we identify and enumerate the member states, civil society groups and industry actors participating in CCNFSDU standard-setting processes. Second, we examine how these stakeholders frame and contest three major revisions to the Standard on FUF, including those on labelling requirements; maximum and minimum protein levels; and permitted levels of free sugars.

## Methods

Given the complex multi-variable nature of the topic under investigation, a case study design and mixed methods approach was adopted, involving quantitative and qualitative components to address the respective study aims.^57^

###  Identification and Enumeration of Stakeholders – Quantitative Component

To address the first study aim, we used descriptive statistics to enumerate and categorize actors who participated at CCNSFDU meetings between 2015 and 2019.^58^ This involved identifying actors from CCNFSDU meeting reports, and distinguishing between those who participated as part of member state delegations (Table S1 and Table S2; see Supplementary files 1 and 2), and those as independent observers (Table S3, Supplementary file 3). Codex categorized observers as UN, IGO, or NGO (independent of governments, generally non-profit, but can receive funding from private sources under the guise of public interest).^59^ We categorized observers by the type of organization they represented; Government (nations not yet members of Codex)^29^; industry (including industry-sponsored consumer organizations)^29^; NGOs (independent of government and industry groups)^59^; and IGOs (entities created via treaty between two, or more nations to work on an issue of common interest with the financial and political support of its member states).^59,60^

Meeting reports were then examined to identify who participated each year.^61-65^ All CCNFSDU attendees were categorized by actor group including, member state, industry, IGO, or civil society (non-industry funded NGOs),^45,53^ and then further into sub-groups, for example, member state delegates by government ministry (eg, health, agriculture, commerce and trade). Member state delegations were categorized as either LIC, LMIC, upper-middle-income country (UMIC), or high-income country (HIC) using the World Bank country income classifications.^66^ The number of individual representatives within each group and sub-group were enumerated for each year, and described as a mean, or percentage of total representatives.^67^

###  Document Search Process and Framing Analysis – Qualitative Component

 To address the second study aim, we adopted a qualitative method involving two steps, a search for relevant Codex documents, and a theoretically guided framing analysis.

The document search process is shown in Figure S1 as a PRISMA flow diagram (see Supplementary file 4). Table S4 (Supplementary file 4) shows the inclusion and exclusion criteria applied. Initial documents were extracted from annual CCNFSDU meeting documents available from the Codex website, including pWG and eWG reports, agendas, reviews, comments and proposals.^61-65^ The websites of stakeholders identified in these reports were also searched for relevant Codex submissions and comments. This generated documents from the International Baby Foods Action Network (IBFAN) (civil society perspective), and United States Department of Agriculture (HIC perspective).^54,68-75^ No further publicly available documents were found. The discovered documents were sorted according to the relevant period. All documents were then read by the lead author, and a list of all standard-setting issues contested by meeting stakeholders were identified. From this list, the three most contested issues (labelling requirements, protein content, and free sugars) were selected for ongoing focus of the study. This was based on opposing comments made by member states delegates and observers, and then approved by group discussion. By including these examples, common themes across standard-setting processes could be identified. Furthermore, the proposal for the revision of the FUF standard was first submitted by NZ in 2010; however, the main discussions regarding these contested aspects took place between 2015 and 2019,^48^ and are still under discussion in 2021.

A constructivist approach and framing analysis method was used to understand how actors contested the three selected issues in the FUF Standard.^76,77^ This was guided by the theoretical framework, shown in Table 1, which was initially developed to analyze frames used in political debates concerning the highly-contested issue of obesity.^77,78^ The ‘frame’ is used as the unit of analysis,^79-81^ defined as a central organizing principle that “governs the subjective meaning [assigned] to social events.”^81^ (p. 10-11). Interest groups can deploy frames as ‘weapons of advocacy,’^82^ within political arenas and policy processes, by portraying problems in terms of causality, responsibility, tractability and benefit in ways that generate (or obfuscate) attention, counter the frames used by opponents and mobilize supporters.^79,80,83,84^

**Table 1:** Theoretical Framework Used to Guide the Analysis, Including Coding Prompts

**Dimensions**	**Prompts for Coding**
Interests	What inherent interests were represented by the proposed arguments?
Problem representation	What were the preferences stated by the actor in relation to specific components of the standard? How did the actor portray the problem, and what solutions were proposed? What were the harms or risks associated with action, or with inaction?
Representation of Codex	How were Codex processes and procedures represented in relation to the revision of the standard?
Outcome	What was the outcome of the revision process?

Abbreviation: Codex, Codex Alimentarius Commission.

Given the highly-technical nature of Codex standard-setting processes, we anticipated actors would draw from evidence to support their interpretations of the contested issues (and to counter opponents), while institutionalised norms and processes at Codex may preference (ie, filter in or out) certain types of evidence over others.^85-87^ The framing of food and nutrition policy issues is often described as fragmented, given the complexity of the issues involved, and the frequent involvement of many different actors and interests.^88,89^ Hence, we anticipated divergences in the frames used by different actor groups and sub-groups to interpret and portray each of the three issues.

To organize and categorize the textual data, documents were uploaded to the qualitative analysis software NVivo (QSR International, version 12.5) and coded for each of the three contested aspects of the draft Standard.^90^ The initial coding schema and prompts were developed from the theoretical framework (Table 1), with codes refined through constant comparative analysis over multiple iterations of document coding.^91^ Themes, associations, and any interrelationships were then identified as they emerged, with the final set of themes synthesised into the final results.^90,92^

## Results

 The results are structured to address the two main objectives for this study. The first section enumerates actors participating in the CCNFSDU. The second presents the results of the framing analysis.

###  Enumeration of CCNFSDU Stakeholders

**Figure 1:**
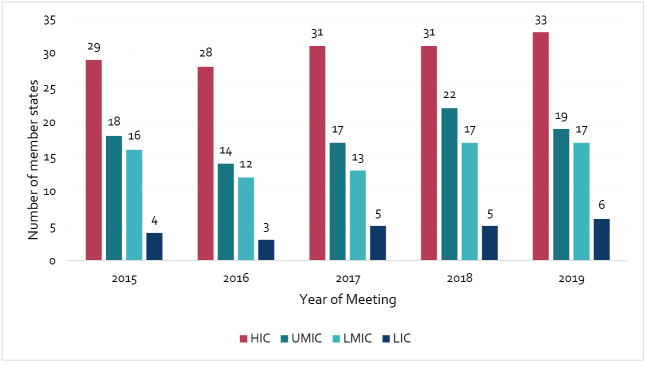
Member State Delegations at CCNFSDU Meetings Based on Country Income (2015-2019). Abbreviations: HIC, high-income country; UMIC, upper-middle-income country; LMIC, low- and middle-income country; LIC, low-income country; CCNFSDU, Codex Committee on Nutrition and Foods for Special Dietary Uses. Notes: Country income classifications are calculated using the World Bank Atlas method and defined as those with a gross national income per capita of $1,035 or less in 2019 for LICs; LMICs, $1,036-$4,045; UMICs, $4,046-$12,535; and HICs, $12,536 or more.^66^

Figure 1 shows the number of member state delegations at CCNFSDU meetings from 2015-2019, grouped by income. Of the 189 Codex member states, there was on average 68 delegations participating each year, over the five years. The number of delegates within each delegation increased with country income status; HICs had the highest mean number and proportion of the total number of delegates (mean = 30.4, 44.7%) and LICs the least (mean = 4.6, 6.8%). The number of delegates and their proportional representation across different income categories remained consistent over the 5-year-period. A mean percentage comparison between member state participation at Codex and the CCNFSDU by country income level is shown in Figure S2 (see Supplementary file 5).

**Figure 2:**
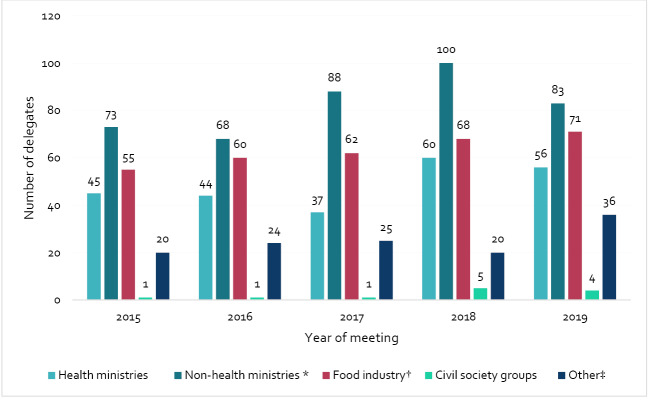
Interest Group Participation at CCNFSDU Meetings as Part of Country Delegations Between 2015 and 2019. Abbreviation: CCNFSDU, Codex Committee on Nutrition and Foods for Special Dietary Uses. *Non-health ministries: represent various non-health government ministries, including agriculture, commerce, trade, food safety, food standards and food and drug administration. †Food industry: represents breast milk substitute companies, the dairy industry, other food industries, and associations representing manufacturers and industry-sponsored consumer organizations. ‡Other: represents embassy personnel, national Codex representatives, academics, hospital personnel, lawyers, and undisclosed delegates.

We found diverse government sectors represented within member state delegations, but also strong representation of the food industry. Figure 2 shows the different interest groups represented across all member state delegations at annual CCNFSDU meetings over the period. Each delegation holds one vote for the adoption, or rejection of a proposal^29^ and any stakeholder within a delegation may have the right to vote when substituting for the country representative, yet observers do not have voting rights.^33,46-51^ Moreover, the Chair facilitates consensus building which is prioritized over voting.^51^ Voting only takes place when consensus cannot be reached. Between 2015 and 2019, the proportion of delegates representing different interest groups remained consistent. The plurality of member state delegates was from non-health ministries (mean = 82.4, 37.2%), followed by the food industry (mean = 63.2, 28.5%), health ministries (mean = 48.4, 21.9%), other (ie, academics, lawyers, unknown etc) (mean = 25, 11.3%) and civil society groups (mean = 2.4, 1.1%). There was an increase in the total number of industry delegates over time, growing from 55 to 71. Additionally, 74 countries sent national delegations with at least half of their delegates from industry. In at least four delegations, a food industry representative was the only member state delegate.

**Figure 3:**
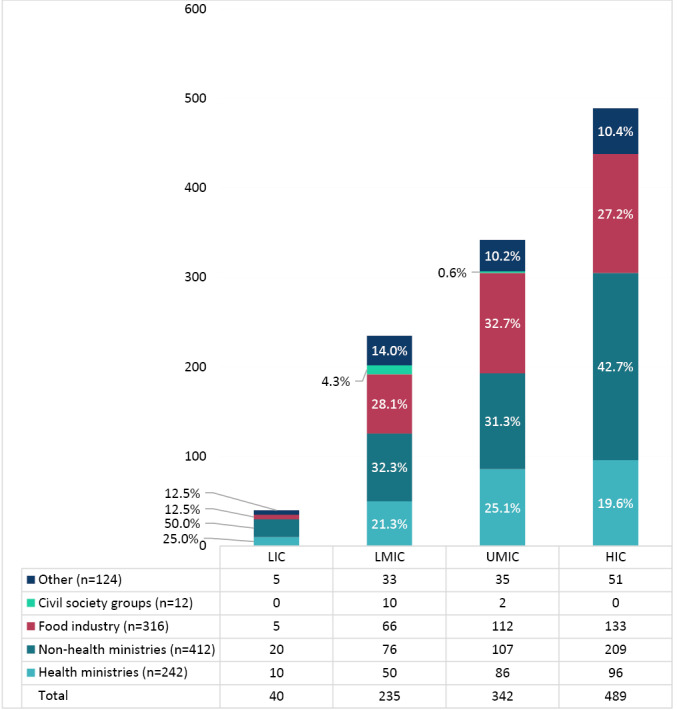
Proportion of Interest Groups at CCNFSDU Meetings Representing Member States by Country Income (2015-2019). Abbreviations: LIC, low-income country; LMIC, lower-middle-income country; UMIC, upper-middle-income country; HIC, high-income country; CCNFSDU, Codex Committee on Nutrition and Foods for Special Dietary Uses.

Figure 3 shows interest group representation in member state delegations, over the five-year period, organized by country income category. LMIC, UMIC and HIC delegations had greater industry and non-health ministry representation compared to health ministry representation, while LIC delegations were more strongly represented by non-health ministries. Civil society groups were least represented in member state delegations.

**Figure 4:**
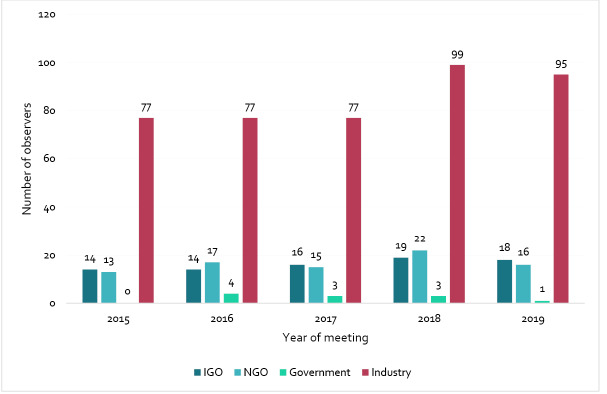
Category of Observers at CCNFSDU Meetings (2015-2019). Abbreviations: IGO, intergovernmental organizations; NGO, non-governmental organizations; CCNFSDU, Codex Committee on Nutrition and Foods for Special Dietary Uses.

Figure 4 shows the number of observers participating in CCNFSDU meetings by interest group. Between 2015 and 2019 the percentage of industry representation was significantly higher (70.8%) than all other observer categories including, NGOs (13.8%), IGOs (13.5%), and government (1.8%). A significant increase in industry participation as observers was seen between 2017 (n = 77) and 2018 (n = 99). Over the five-year period, there was a minor increase in the number of IGO and NGO observers.

###  Thematic Analysis of Three Contested Aspects of the Draft for Follow-up Formula

The following section presents the results of the framing analysis, organized by the three issues contested during the FUF standard-setting processes – labelling requirements, protein composition, and free sugar content. These issues attracted considerable discussion among stakeholders during CCNFSDU meetings. Table 2 shows the stakeholders involved in contesting each of the three issues, a summation of the primary and secondary frames they deployed during the Standard revision process, and the interests represented by these frames including public health interest/pro-health interest (supporting global health and/or breastfeeding), commercial interest/pro-commercial interest (supporting industry growth, and/or trade facilitation), and mixed interest (supporting a combination of both). In the following sections, further detail is provided of the pro-health and pro-commercial frames used to contest each issue, and the final deliberations and outcome for the draft revision.

**Table 2:** Frames Used to Contest Labelling Requirements, Protein Composition, and Addition of Free Sugars in the Standard on Follow-up Formula

**Issue (and Interests)**	**CCNFSDU Stakeholders**	**Primary Frame**	**Secondary Frame**
**Labelling Requirements**			
Public health interest	Member state delegations (no industry delegates): Botswana, Burkina Faso, Cambodia, Ecuador, India, Kuwait, Lao People’s Democratic Republic, Mali, Niger, Senegal, Sri Lanka, Uruguay. Member state delegations (with at least one industry delegate): Chile, Mexico, Nigeria, Norway, Sudan, Switzerland. Member state delegates (with at least one civil society group delegate): Nepal, Cambodia, Lao People’s Democratic Republic, United Republic of Tanzania. Observers: IBFAN, WPHNA, UNICEF.	• Cross-promotion practices should be prohibited between Infant and FUF products according to WHO guidelines and WHA resolutions, including *The Code*.	• Cross-promotion practices should be prohibited to align with WHO guidance and *The Code* to end inappropriate marketing of BMS and support, protect and promote breastfeeding. • Cross-promotion leads to consumer confusion and the wrong product being used for infants. • Cross-promotion is a form of marketing and commercial strategy that compromises consumers, eg, brand extension and product line extension. • Cross-promotion has a negative impact on the health and development of infants and children, particularly in developing countries. • Trade and commerce interests created by cross-promotion should not be prioritised over health. • It is important for all delegation and observer positions on cross-promotion to be discussed and considered.
		• Labelling requirements for all FUF products should be aligned.	• Labelling requirements for all FUF products should be aligned for consistency and legal clarity.
Commercial interest	Member state delegations (no industry delegates): Cuba, EU, Guatemala, Republic of Korea, Panama, Peru, South Africa, Zimbabwe.	• Reference should not be made to the WHA resolution 69.9 within the FUF Standard.	• WHA resolution 69.9 was technically not approved by the WHA, but “welcomed with appreciation.” Therefore, it did not belong in the revised Codex FUF Standard.
	Member state delegations (with at least one industry delegate): Argentina, Australia, Brazil, Canada, People’s Republic of China, Costa Rica, France, Indonesia, Japan, Malaysia, NZ, Russian Federation, Thailand, US, Viet Nam.	• WHO policy documents should not be referred to in the FUF Standard.	• WHO policy documents are outside the scope and mandate of Codex, could create political and legal consequences, and undermine the credibility of the standard.
	Member state delegations (with at least one civil society group delegate): Brazil. Observers: AEDA, IDF/FIL, ISDI.	• “Cross-promotion” should not be included in the text for the FUF Standard.	• There is no clear Codex definition of “cross-promotion.” • Including the phrase “cross-promotion” in the text of the standard could create unnecessary barriers to commerce and trade. • Inclusion of text covering text, images and colors on labels is subjective and open to different interpretations. • Alternate text should be used to describe marketing restrictions.
		• Labelling requirements for FUF should be flexible.	• Labelling requirements for both sections of the Follow-up- Formula Standard should not be more stringent, prescriptive, or restrictive than the IF Standard.
Mixed interest	Member state delegations (no industry delegates): Botswana, Burkina Faso, Cambodia, Ecuador, EU, Mali, Niger, Peru, Senegal. Member state delegations (with at least one industry delegate): Australia, Costa Rica, Indonesia, Kenya, NZ, Norway, Switzerland. Member state delegations (with at least one civil society group delegate): Brazil, Cambodia, Nepal.	• The intent of “cross-promotion” is important, (ie, it should not discourage breastfeeding, and products should be distinguishable) but not the proposed text in the FUF Standard. Therefore, the removal of “cross-promotion” in the “spirit of compromise” is the appropriate option.	• To allow work on the draft to progress.
**Protein Composition**			
Public health interest	Member state delegations (no industry delegates): EU, United Republic of Tanzania. Member state delegations (with at least one industry delegate): Canada, Costa Rica, Kenya, Norway. Member state delegations (with at least one civil society group delegate): United Republic of Tanzania.	• Not supportive of lower minimum protein levels in FUF.	• Lower minimum levels of protein, watered-down products, and inadequate local sources of protein during weaning, contribute to insufficient protein intake and malnutrition. • The lower minimum protein level was based on research on European infants which is not relevant to countries where protein quality is poor, and intake is low.
	Observers: IBFAN.	• Supportive of lower maximum protein levels in FUF.	• Lower protein levels can reduce renal loads and avoid associated risks of high protein intakes.
		• Health considerations should be preferred over trade considerations when determining appropriate protein levels in FUF.	• Trade considerations taking precedence over health considerations is not acceptable.
		• Lower minimum protein levels should not be assessed, or “scientifically substantiated” by manufacturers, or distributors.	• The “scientific substantiated” assessment of minimum protein levels can lead to industry sponsored research and inappropriate composition of FUF.
Commercial interest	Member state delegations (no industry delegates): EU. Member state delegations (with at least one industry delegate): Canada, Chile.	• The minimum protein level in FUF should be higher.	• The higher minimum protein level will support neurodevelopment, growth and maintenance, and global requirements.
	Observers: ISDI, IDF/FIL.	• The minimum protein level in FUF should be lower.	• Early Nutrition Academy recommendations indicate the lower minimum level is sufficient to cover maintenance and growth. • If EFSA scientific evidence supports a lower minimum protein level, then it should be adopted.
		• Lowering the maximum protein level from the level in the original FUF Standard should be transitioned over time.	• Lowering maximum protein levels could cause significant issues for trade.
		• The wording “clinically evaluated” in the footnote for lower minimum protein levels should be removed and replaced with “scientifically substantiated.”	• National authorities should assess protein requirements based on local diets as presented by manufacturers.
Commercial dairy interest	Member state delegations (no industry delegates): EU. Member state delegations (with at least one industry delegate): NZ, Thailand, US, Philippines. Observers: IDF/FIL.	• The higher maximum protein level is safe and suitable in FUF.	• IGO guidelines support milk as being part of a healthy diet for young children, and safe for older infants. • The maximum protein level should be based on the minimum level required for energy balance and the deposition of tissues consistent with good health.
		• Minimum protein levels in FUF should be based on the amount needed for nitrogen equilibrium and the levels present in cow’s milk.	• Minimum protein levels should be consistent with good health and energy balance.
Other commercial interest	Member state delegations (no industry delegates): None. Member state delegations (with at least one industry. delegate): Egypt.	• Supportive of lower minimum and lower maximum protein level (soy, cow’s and goat’s) in FUF.	• By default, this would raise the carbohydrate ratio and allow for added sugars.
	Observers: ENSA, EUVPRO, AOCS, ISDI, ELC.	• Maximum protein levels in FUF should not be lowered.	• If maximum protein levels are too low this could increase the carbohydrate ratio.
		• “Clinically evaluated” should be removed from the existing FUF Standard footnote and replaced with “scientifically substantiated.”	• Protein levels that are “scientifically substantiated” are more appropriate than assessing nutritional requirements via clinical trials, particularly in developing countries where trials are rarely conducted.
		• National authorities should not assess FUF protein requirements.	• Manufacturers should be able to assess protein requirements based on scientific-evidence and local diets.
Mixed interest	Member state delegations (with at least one industry delegate): Canada.	• Supportive of a higher minimum protein level in FUF.	• The higher minimum protein level is suitable for most regions based on the EFSA report.
	Member state delegations (no industry delegates): EU.	• Supportive of a lower minimum protein level in FUF.	• To support nutritional considerations for developing countries.
		• Supportive of a lower maximum protein level in FUF.	• To avoid an increased burden of disease. However, this could create significant issues for trade compliance if transitioned too quickly.
**Addition of Free Sugars**			
Public health interest	Member state delegations (no industry delegates): Burkina Faso, Ecuador, EU, Guatemala, India, Lao PDR, Mali, Panama, Sri Lanka, South Korea, United Republic of Tanzania. Member state delegations (with at least one industry delegate): Brazil, Canada, Kenya, Mexico, Morocco, Nigeria, Norway, Switzerland.	• Sweet-tasting additives in FUF should be limited.	• Limiting sugar intake, particularly sweet tasting additives, will reduce overweight, obesity, other NCDs, dental caries, and conditioning towards sweet tastes.
	Member state delegations (with at least one civil society group delegate): Brazil, United Republic of Tanzania. Observers: HKI, IDF/FIL, WPHNA.	• Available carbohydrates (other than lactose) should be limited to 5-10% in FUF.	• Limiting available carbohydrates is the best choice in countering several global health issues. • Consumption of FUF with high carbohydrate levels that do not fall within WHO sugar-intake guidelines, can cause excessive energy intakes.
		• Future alternate carbohydrate sources added to FUF could impart or enhance a sweet taste.	• The text in the standard should support the prohibition of possible technological innovations of sweet-tasting non-sugar ingredients in future FUF products.
Commercial interest		• The text should not state fructose and/or sucrose can be added to FUF “if needed.”	• Inclusion of text allowing fructose and/or sucrose to be added “if needed” is contradictory to WHO sugar-intake guidelines and provides a pathway towards obesity and NCDs.
	Member state delegations (no industry delegates): EU.	• The maximum carbohydrate level in FUF should be flexible.	• Flexible maximum carbohydrate levels allow for FUF to contain lower levels of protein and fat.
		• Scientific evidence by an international expert group, including the Early Nutrition Academy, does not support a lower carbohydrate level for young children.	• 14 g/100 kcal of available carbohydrates represents less than one teaspoon of sugar in 200 ml of FUF.
	Member state delegations (with at least one industry delegate): Argentina, Canada, Chili, Colombia, Costa Rica, Japan, Russia, Canada, Australia, NZ, US. Observers: EUVPRO, IDF/FIL, ISDI.	• Sweet-tasting additives should not be restricted, therefore, the sentence prohibiting fructose and/or sucrose should be removed from the Follow-up- Formula Standard. • The sentence in the text referring to “sweet-tasting” should be removed from the FUF Standard.	• Other mono- and disaccharides and/or glucose polymers should be permitted and there is no scientific evidence to support the exclusion of sucrose and fructose in FUF. • The phrase “sweet-tasting” is subjective, lacks definition, and is not enforceable.
		• The text in the FUF Standard should protect against technological innovations in sweeteners.	• Future multi-purpose non-caloric sweeteners may be added to FUF if the Standard is not “future-proofed.”
		• The text in the FUF Standard should provide the opportunity for sweet-tasting additives for future inclusion even if they do not have a nutritional purpose.	• It would be constraining to include wording that prohibits future non-caloric or artificial sweeteners.
		• Overall, the provisions for carbohydrates in FUF are too restrictive.	• Provisions for carbohydrates should be aligned with existing national regulations and the standard for IF and FUF for older infants.
Mixed interest	Member state delegations (no industry delegates): EU. Member state delegations (with at least one industry delegate): Canada, Colombia.	• Corn maltodextrin should be included as a carbohydrate source in soy-based FUF.	• Corn maltodextrin addition to soy-based FUF is in accordance with existing North American regulations.
	Observers: IDF/FIL.	• A lower maximum carbohydrate level of 1.25 g/100 kcal is preferred in FUF, along with a 10% limit for added sugars (excluding lactose).	• Sweet-tasting FUF and high carbohydrate levels (other than lactose) can contribute to diabetes mellitus, cardiovascular disease and long-term weight gain as shown by scientific evidence. • Lactose is strongly supported as the preferred carbohydrate.

Abbreviations: AEDA, European Food Law Association; AOCS, American Oil Chemists Society; ELC, Federation of European Specialty Food Ingredients Industry; EFSA, European Food Safety Authority; ENSA, European Natural Soy and Plant-based Food Manufacturers Association; EU, European Union; EUVPRO, European Vegetable Protein Association; FUF, Follow-up Formula; HKI, Helen Keller International; IBFAN, International Baby Foods Action Network; The Code, International Code of Marketing Breast-milk Substitutes; IDF/FIL, International Dairy Federation; ISDI, International Special Dietary Food Industries; NCD, non-communicable disease; UNICEF, United Nations Children’s Fund; US, United States; WHA, World Health Assembly; WHO, World Health Organization; WPHNA, World Public Health Nutrition Association; Codex, Codex Alimentarius Commission; CCNFSDU, Codex Committee on Nutrition and Foods for Special Dietary Uses; IGO, ntergovernmental organization; BMS, breast-milk substitutes; NZ, New Zealand; IF, infant formula. Notes: Public health interest delegations were determined based on arguments/proposals supporting global health and/or breastfeeding; commercial interest delegations were determined based on arguments/proposals supporting industry growth, and/or trade facilitation; and mixed interest delegations were determined based on a combination of both arguments/proposals.

###  Labelling Requirements

 The most contested revisions to the FUF standard concerned labelling requirements, covering marketing techniques and product label information, particularly, the integration of text concerning “cross-promotion,” based on the 2016 WHO technical guidance, and Resolution WHA69.9.

*Pro-health interest frames:* During eWG consultations, some LMIC delegates expressed concerns that FUF labelling was being used inappropriately to cross-promote IF. Kenya and Tanzania mentioned similar labelling caused the accidental consumption of formulas that were unsuitable for infants. Helen Keller International (HKI) presented evidence from the Assessment and Research on Child Feeding Project^23^ showing that infant, follow-up, and toddler-milks were frequently cross-promoted in Cambodia, Nepal, Senegal and Tanzania. For example:

“*…many manufacturers commonly use a No 1 on the labels of their infant formula, a No 2 on their follow-up formula for older infants 6 -12 months and a No 3 on their product for the young child 12-36 months. Although different companies might take a slightly different approach, they clearly position these as a single category made of sub-sets and all are breastmilk substitutes*” (HKI, 2016).

Subsequently, the CCNSFDU considered revisions to the standard prohibiting cross-promotion between product categories, such as using the same brand name, or label design as IF. India, Nigeria, and Sudan supported this amendment, stating that cross-promotion negatively impacted infant and young child health. In 2016, consideration was given to add the aforementioned WHO technical guidance and WHA69.9 to the scope, or labelling section of the revised FUF standard. Several delegations, including Mexico and China, expressed support for this proposal. IBFAN emphasised that marketing restrictions covering all products for ages 0-36 months, as outlined in *The Code* and subsequent resolutions, would reinforce restrictions on BMS promotion. Cambodia was concerned that, “*Codex plays a critical role in protecting optimal infant and child feeding practises…[yet] trade and commercial interests are clearly taking precedence over health*” (Cambodia, 2018).

 In agreement with the European Commission regulations, the EU consistently reiterated that labelling was not to discourage breastfeeding, but to ensure products are “clearly distinguishable,” with the standard defining “text, images and colours used” (EU, 2017). The Committee agreed and introduced a statement prohibiting any form of cross-promotion from appearing on labels and added young children to the list of prohibited pictures which included women, infants, and older infants.

 For “legal clarity,” IBFAN recommended, the labelling provisions for all products labelled as FUF must be aligned, and denounced how the CCNFSDU conducted proceedings, saying it:

“…*failed to allow sufficient discussion and consideration of the positions of all member states regarding cross-promotion, marketing and labelling of these products. The products are highly likely to be confused with infant formula and their global trade risks the replacement of breastfeeding and the undermining of child health*” (IBFAN, 2019).

*Pro-commercial interest frames: * The EU, France, and US contested inclusion of Resolution WHA69.9 claiming that the WHA “welcomed with appreciation,” but “did not approve or endorse the WHA resolution” (EU, France, and US, 2017). These stakeholders commented on technicalities with the language used by the WHO. The US and the European Food Law Association noted that WHO policy documents were outside the scope and mandate of Codex, could create political and legal consequences, and undermine the relevance and credibility of these standards. Inclusion of WHA69.9 was interrupted pending inquiries to the WHO legal office.

Ignoring WHO definitions and the General Standard for the Labelling of Pre-packaged Foods, which states labelling should not suggest a connection to other products or cause confusion between products,^35^ the US stated that within Codex texts, there was no definition for “cross-promotion” and warned the term could cause unnecessary barriers to trade. The EU, Argentina, NZ and International Special Dietary Food Industries (ISDI) suggested the prohibition would make the standard excessively “prescriptive” (EU, 2016 and Argentina, NZ, and ISDI, 2017). Overall, pro-commercial delegates considered “inclusion of text covering text, images and colors used is very subjective and open to different interpretations” (ISDI, 2017).

*Mixed interest frames: * In 2019, after the Codex Committee of Food Labelling aligned with pro-commercial stakeholders in reference to Codex having no clear definition for “cross-promotion,” Australia proposed alternate text, and the Chair requested that stakeholders further discuss this detail. This led to delegations (Botswana, Niger, Senegal, Mali, Switzerland, Kenya, Cambodia, Burkina Faso, Nepal, Norway, and Ecuador) changing their position from endorsing the prohibition of “cross-promotion” to agreeing to its removal. Previously, Burkina Faso described cross-promotion as “purely and simply, a commercial strategy that compromises consumers” (Burkina Faso, 2019). Similarly, Mali had argued that “cross-promotion” through FUF was “a well-established business strategy” used to promote IF (Mali, 2019). The group agreed to strike the prohibition “in the spirit of compromise.”

*Final deliberations and outcome:* The CCNFSDU approved the removal of the term “cross-promotion” and prohibit cross-promotional images, text, statements, or numbers for FUF-OI. The CCNFSDU did not agree to seek a definition for “cross-promotion” from WHA 63.14, WHA 69.7 or other WHO documents, to be included in the standard.

###  Protein Composition

Normal growth and development for older infants and young children, requires appropriate protein intake, and therefore, establishing minimum and maximum requirements was an important consideration in the composition of FUF.^93^ However, in accordance with the original Standard for FUF, the protein reference intake values were found to have been overestimated at 3.0-5.5 g/100 kcal,^17,93-96^ and required a reduction to circumvent obesity and associated diseases.^93^ Consequently, research was conducted by the European Food Safety Authority (EFSA),^97,98^ to establish appropriate protein levels and inform Codex FUF standard revisions.

 Several protein level considerations were contested during CCNFSDU proceedings, including appropriate minimum and maximum levels, whether to base levels on regional requirements, and whether protein content should be evaluated by national/regional authorities, or manufacturers.

*Pro-health interest frames:* Several country delegations, particularly LMICs, voiced concerns that low minimum protein levels were potentially harmful to public health. Kenya and Tanzania described an already “high incidence of protein and energy malnutrition” during weaning in regions where FUF products were watered-down and local complimentary foods were low in protein content (CCNFSDU, 2017). Canada promoted the higher minimum protein level with a lower option for countries with minimal requirements.

 Other pro-health frames included protection of population health when promoting lower maximum protein levels. Norway argued this could, “avoid potential risks associated with high protein intakes” and Costa Rica said, “it does not represent an excess renal load” (Norway, 2015 and Costa Rica, 2016). Both reflected the EU Commission’s delegated regulations and EFSA findings. Although, EFSA findings were criticised by IBFAN as being “wholly inappropriate to infants in other parts of the world” (IBFAN, 2016).

*Pro-commercial interest frames: * Most pWG members and countries associated with the dairy industry, argued that a higher minimum protein level was necessary for neurodevelopment, growth, and maintenance. Some industry groups consistently presented scientific evidence to frame arguments for, or against specific protein levels. For example, ISDI argued that 1.65 g/100 kcal was “the protein minimum requirement (is) set to cover maintenance and growth” based on Early Nutrition Academy recommendations (ISDI, 2015), which was agreed to by some pWG members if supported by the EFSA report.

 Industry observers were split on the issue with dairy suppliers diverging from other FUF manufacturers. Dairy producers frequently referred to IGO guidance to support higher protein allowances. For example, IDF/FIL argued that “milk is recognized as an important part of a healthy diet for young children,” a statement previously made by the FAO, before proposing the higher maximum protein level as “safe and suitable for consumption by older infants, [and] has a long history of apparent safe use.” This was supported by WHO recommendations that proposed levels would also accommodate “the deposition of tissues consistent with good health” (IDF/FIL, 2016). Comparatively, Egypt, who had high BMS manufacturer and beverage industry representation (Nestlé, PepsiCo and Coca-Cola) agreed with lower protein levels for soy, cow’s, and goat’s protein. This concerned other member states because lowering maximum protein levels would significantly increase the carbohydrate ratio, allowing for added sugars.

 Other industry groups based arguments on scientific evidence, prompting support from LMICs and UMICs. For instance, Vietnam and Malaysia agreed with ISDI recommendations to change “clinically evaluated” to “scientifically substantiated” in a footnote for lower minimum protein levels, which could allow for industry-sponsored research to influence FUF composition. Similarly, Argentina, Malaysia and Vietnam agreed with ISDI’s suggestion that manufacturers present to national authorities their assessment of local protein requirements. This was disputed by IBFAN who stressed that assessments should be independent of manufacturers and distributors.

*Mixed interest frames: * Canada simultaneously noted potential trade and public health concerns, recommending a transition period to support eWG concerns that reduced protein composition could cause significant issues for trade compliance and generate the risk of trade barriers, but disagreed with higher maximum protein levels because, “high infant milk protein intakes during the first year of life that markedly exceed metabolic requirements were shown to lead to excessive weight gain which can increase the risk of later obesity and associated diseases” (Canada, 2015). Trade considerations taking precedence over health was “not acceptable,” according to IBFAN (2015). The eWG recommendation was agreed to by both LMICs and HICs with a transition period for industry to adapt to the lower maximum level.

*Final deliberations and outcomes: * The Committee agreed to higher minimum protein levels for FUF-OI and FUF-YC of 1.8 g/100 kcal which was aligned with the positions of 13 countries and 1 member organization (with voting rights), and with those of 5 observers (without voting rights). The Committee approved the lower 3.0 g/100 kcal maximum for FUF-OI when consensus was not reached to align with the IF Standard, however this was the preferred level of 11 countries and one member organization. A footnote was also approved to stipulate the clinical evaluation for safety and suitability, by competent national and/or regional authorities.

###  Addition of Free Sugars

To meet carbohydrate energy density requirements for older infants and young children, the original Codex Standard for FUF recommended 60-85 kcal/100 mL.^17^ However, WHO guidance and updated scientific research on sugar intake have since determined this range to be markedly higher than breastmilk, and may increase health risks, such as excess weight gain and dental carries.^48,99^ Consequently, CCNFSDU deliberations focused on maximum available carbohydrate levels, the addition of “sweet-tasting” free sugars; fructose, sucrose, and sweeteners other than lactose, and “future-proofing” the standard.

*Pro-health interest frames:* Early in discussions, pro-health stakeholders agreed that sweetened FUF products contributed to obesity and non-communicable diseases (NCDs), and limiting available carbohydrates other than lactose was important. Brazil, Norway, Switzerland and HKI agreed that limiting free sugars to 10% of available carbohydrates was “the best choice in countering several health issues that are rapidly increasing worldwide” (Switzerland, 2017). A WHO representative explained, “consumption of 300ml to 500 mL of follow-up formula…would provide 20-33% of energy requirements in the diet of young children,” and to remain within WHO guidelines “products would need to contain less than 8g of added sugars” (WHO, 2016). The WHO representative argued that text allowing sucrose and/or fructose to be added “if needed” was contradictory to these guidelines.

 Altogether, 11 member states, two civil society groups and one IGO, advocated for limiting sweet-tasting additives with Canada warning that “the seeds of obesity are sown in early childhood when the preference for sugar-rich sweet-tasting foods and drinks is established” (Canada, 2018). This led to discussions about alternate carbohydrate sources and several member states voicing concerns over “future technological innovations” that could be added as ingredients to impart or enhance a sweet taste in FUF-YC (Brazil, 2019).

 Civil society group HKI, addressed “future-proofing” FUF products as:

*“…a critical issue as the world increasingly faces and is required to address the issue of overweight and obesity in children – it is estimated that by 2030 250 million children worldwide will be obese – and that the period 12-36 months is critical in ensuring children do not become conditioned to sweet tastes”* (HKI, 2019).

*Pro-commercial interest frames:* Exporting member states with strong industry representation were generally opposed to restricting sweet-tasting additives. Six member countries and one industry stakeholder referenced recommendations published by an international expert group (in collaboration with BMS companies)^94^ when proposing a higher carbohydrate limit (14.0 g/100 kcal), which was argued as representing less than one teaspoon of sugar in 200 ml of formula. To accommodate proposals for lower fat and protein limits, NZ recommended flexible carbohydrate levels. Consequently, several pro-commercial countries voted for a footnote for a higher carbohydrate limit for products containing less than 3.0 g/100 kcal of protein when permitted by local authorities.

 Existing national regulations were used by NZ, Argentina, Canada and ISDI to favour consistency with the Standard for IF. Australia referenced the Chair’s comment that limiting sugars in FUF-YC was more restrictive than IF and FUF-OI, and along with NZ, argued for the deletion of the sentence prohibiting fructose and/or sucrose. The US argued:

*“…For non-milk-based products which tend to have a bitter taste, use of other mono and disaccharides and/or glucose polymers should be permitted within the carbohydrate level constraints…and we are not aware of a scientific basis for the exclusion of sucrose and/or fructose”* (US, 2019).

 Australia, Brazil, Canada, Mali, Sri Lanka and HKI used “future-proofing” as a frame to accommodate possible technological innovations such as “the situation where ingredients may have multiple purposes” (Australia, 2019). Other industry representatives and the US noted that “sweet taste is subjective, has no definition, and is not enforceable” and future noncaloric, or artificial sweetener-type ingredients would be “constrained” by nutritional purpose requirements. (US, 2019).

*Mixed interest frames: * Scientific evidence was referenced by IDF/FIL who was in favour of a 10% limit for added sugars (excluding lactose), “strongly” supported lactose as the preferred carbohydrate, and warned that refined sugars negatively impact insulin sensitivity associated with diabetes mellitus, cardiovascular disease, and long-term weight gain. Conversely, in alignment with North American regulations, Canada recommended corn maltodextrin as an alternative for soy-based formulas, providing it does not exceed 10% of available carbohydrates.

*Final deliberations and outcomes:* By the close of 2019 the Committee had agreed that the preferred carbohydrate was lactose, and free sugar limits were set to reduce any sweet taste: the agreed carbohydrate range for FUF-OI was 9.0-14.0 g/100 kcal with sucrose and/or fructose (if needed) to not exceed 20%; the agreed range for FUF-YC was 12.5 g/100 kcal, sucrose and/or fructose were prohibited, and sources of available carbohydrates (other than lactose) could not exceed 2.5 g/100 kcal.

## Discussion

 This study aimed to enumerate stakeholder participation at Codex meetings, understand how interests are promoted, and determine how contested aspects of the proposed draft for the FUF standard for infants and young children are framed.

We showed clear trends in participation by delegates (with voting power) and observers at CCNFSDU meetings between 2015 and 2019. Within member state delegations, the higher the country income, the greater the number of delegates, which meant that LICs (who arguably are most vulnerable to the harms of misuse of FUF) were poorly represented. In addition, the wealthier the country, the greater the number of industry and non-health ministry representation. Notably, the largest exporters of BMS and other relevant products, such as dry milk powder, are among the HICs.^100^ The absence of civil society group representatives in HICs and LICs suggests that their voices were underrepresented. Overall, there was much stronger representation of industry over civil society group delegates and non-health ministry over health ministry delegates at CCNFSDU meetings during the five-year period. However, it is important to note the uncertainty in who influenced comments, as was evident when Kenya expressed concerns about cross-promotion while represented by industry and government ministries in 2017, and in 2019 when Burkina Faso and Mali changed their stance on retaining “cross-promotion” in the standard while represented by government ministries. In this case, it is unclear whether delegates were influenced by the Codex Committee of Food Labelling, the Australian proposal, or other participants. The composition of delegates within delegations changed from year-to-year. Although, submissions appeared to be influenced by stakeholder groupings within a delegation with the most representatives.

 As observers, industry presence was also disproportionately high compared to NGO, IGO and government attendance.

 The qualitative results showed a marked difference in framing between countries that prioritise health, those that favour commerce and trade, and those with dual interests while debating labelling requirements, protein content and the addition of free sugars. This was also evident when comparing civil society groups to the private sector.

When contesting BMS labelling requirements, public health interest actors framed arguments in favour of protecting breastfeeding; prevention of harms caused by cross-promotion; and infant, child, and maternal health. In contrast, commercial interest stakeholders used legal and technical loopholes to avoid integrating *The Code*, and WHA69.9 into the Standard for FUF, which would reinforce restrictions on marketing practices. Most, framed arguments to prevent barriers to commerce or trade. This was favoured by countries home to industries that benefit from BMS promotion.

 Deliberations over protein content showed how public health advocates focused on the impact decision-making and research would have on global health, such as malnutrition, neurodevelopment, growth patterns and NCDs. However, commercial arguments frequently protected trade interests, advocated for FUF protein levels favourable to industry, or interests of exporting countries, and served to preserve nutrient recommendations based on industry-driven research. Some member states, such as Canada and the EU, moved between health and trade interests to protect at various times both public health, and industry interests in international commerce, while encouraging national/regional autonomy.

 While discussing the addition of sweet-tasting free sugars, the overall message shared by public health actors was that reducing child preferences for sweet tastes, prioritising inclusion of lactose, and “future-proofing” the FUF Standard against the emergence of noncaloric or artificial sweeteners, could effectively decrease NCDs. However, commercial stakeholders regularly supported proposals targeting technical loopholes to obfuscate public health interests and protect commercial interests for the inclusion of new sweeteners. Frames used often looked to national regulations or were based on a narrow set of scientific studies, as opposed to studies of population risk of NCDs, or WHO guidelines. Some countries engaged with multiple frames reflecting national, or regional commercial interests.

These findings are similar to those of Thow et al,^33^ Arendt^46^ and Koletzko et al^47,48,101^ and other commentaries^50,102-105^ that describe the Codex standard-setting processes as: (*i*) heavily influenced by industry stakeholders; (*ii*) regulated by a governance regime of closely connected organizations and influential member states; and (*iii*) largely based on industry citing poor quality studies ‘cherry picking’ evidence. The findings further demonstrate that the presence of civil society groups is essential in representing the welfare of infants and young children and in keeping *The Code* and WHA69.9 central to Codex deliberations.

The results indicate that more public health, both in national delegations and among observers, and LIC, and LMIC representation is needed. Currently, the Codex regulatory process is influenced by internal and external politics which have intensified with the growing involvement of industry and pressure of facing trade challenges under WTO agreements.^106^ This creates obstacles in standard-setting processes which are contrary to the transparency and inclusiveness that Codex claims to uphold.^38^ While Codex and the WTO agreements encourage active participation by lower-income countries, this relies on thorough preparation and multisector collaboration, along with the establishment and maintenance of technical and institutional capacities.^37,38^ These resources are available to UMICs and HICs but, LICs and LMICs do not have the same capacity.^38^ To address this imbalance, the Codex Trust Fund was established in 2004 with a successor in 2016 to support participation by developing and transition economy countries, including 27 participating and 104 eligible countries.^29,107-109^ However, during the first 12 years, the Codex Trust Fund sought $40 million, yet received only $18 million from 15 member states.^110^ Moreover, the diverse viewpoints demonstrated by LIC and LMICs are important as they lead to meaningful debates, as evident during cross-promotion deliberations. Therefore, further strategies are needed to address under-representation of these groups and power asymmetries between stakeholders.

 Generally, delegations operated as industrialised countries aligning with private sector stakeholders, or countries advocating public health-orientated positions with public health advocacy group delegates from civil society. Occasionally, member states displayed conflicting agendas, protecting both consumer health and trade, as was evident in the debate regarding “cross-promotion.” Overall, this appeared to reflect the ratio of health ministry to non-health ministry representation, or industry and civil society group representation within a delegation on a given year. Minimal LIC, health ministry and civil society group participation, as part of country delegations, and as observers, ensured their limited influence over decision-making. These findings indicate that public health issues, particularly in relation to FUF, may be more strongly considered at CCNFSDU meetings if there was a reduced industry, increased civil society, and well-funded equal participation of country delegations by income. Additionally, this research raises the following question: should industry be qualified to represent member states?

This research shows that a very small number of public health civil society groups represent the interests of mothers, infants, and young children at Codex, in particular HKI and IBFAN. These groups provide crucial support to LMICs in navigating the multilateral negotiations that impact the health and wealth of their nations. This is critical because when appointed as part of a national delegation by a chief country delegate, these groups have voting rights, and help LMICs make position statements at standard-setting meetings which can abate industry group influence to gain endorsement (votes) for their specific interests. Meanwhile, this is achieved with minimal financial assistance. If funding from donations, or private organizations such as, the *Bill *&* Melinda Gates Foundation*, is lost, then support from civil society groups will no longer be available for developing countries. This will diminish their participation at Codex and put global infant and young child health in a precarious position. Additional constructive developments, such as the Codex Trust Fund, would also be of benefit.

Moreover, the engagement of food corporations in sponsoring nutrition research may strongly influence standard-setting processes at Codex. According to Marion Nestle,^111^ industry-funded scientific research represents a conflict-of-interest and serves to undermine global health while threatening scientific integrity. This was evident when a guidance report on FUF compositional requirements, involving the Early Nutrition Academy, was presented at CCNFSDU meetings to strengthen private sector and exporting country rationale for lower protein and higher carbohydrate content. The authors stated they were “strongly biased in favour of breastfeeding,” and the disclosure statement did not declare any conflicts-of-interest.^48^ However the report received funding from leading BMS corporations; Nestlé, Danone, Mead Johnson, Abbott, Pfizer, and others.^48^ This demonstrated that Codex’s protocol in recommending expert advice and research capability outside of JEMNU, was inappropriate and open to conflicts-of-interest which is contradictory to the stringent policies in place “to ensure the excellence, independence and transparency” of FAO/WHO experts^38^ (p. 8).

Furthermore, while many stakeholders claimed to be sympathetic towards breastfeeding, it has not been supported, protected, or promoted rigorously in Codex BMS standards. For instance, in countries where *The Code* has not been adopted (eg, the US and Australia), marketing of FUF and toddler milks is regularly used to promote IF by proxy.^21^ Although this study does not establish causality, this likely may explain the rationale behind many BMS companies and HICs adamantly rejecting any inclusion of WHA resolutions in the text for Codex FUF-OI, and FUF-YC standards. It also explains the drawn-out debate against “cross-promotion” being included in the text. However, the approach taken by these actors was not to simply reject a proposal, but rather to frame it with a positive sentiment (eg, for nutritional considerations). These debates were aggressively persistent and reiterated by other HIC and industry groups in an exercise-of-power. One recent study, assessed IF marketing and the extent of the power, size and resources available to the baby-formula industry and impact it makes on government policy-makers by treating them as markets to be targeted.^21^ The study declared “the results make uncomfortable reading”^21^ (p. 9)and pointed out the “urgent”^21^ (p. 10) need for stronger regulations to challenge the normalisation of IF and its marketing practices. Interviews with industry practitioners also revealed intensification of lobbying by BMS stakeholders in the US prior to WHA meetings.

###  Strengths and Limitations

 This study has contributed to our understanding of the structural arrangements and participation inequities within this powerful public health policy-making setting. The results also provided deep insights into the intricate policy-making processes.

There were several limitations encountered. For instance, it was not possible to access CCNFSDU meeting minutes. This could have filled information gaps and enabled a more nuanced interpretation of the standard-setting process and power plays as they unfolded, such as informal discussions and lobbying, and the role of the Chair in selecting which countries speak and who proposes the consensus. Data collected were also unable to verify some delegates as representing industry, or other organizations. At the 41st CCNFSDU meeting, 11 Vietnam delegates were listed without organization names. A thorough internet search identified five as industry personnel, which increased industry representation to 12 out of the 21 delegates.^61^ Five remained unidentified, therefore, a true reflection of industry representation could not be determined.

 Another limitation was not having access to full information about delegates, including industry stakeholders, who were given voting power by member state chief delegates. Some industry actors representing member states, or participating as observers, could not be identified within associations, or consumer market groups. For example, the research identified that ISDI was represented within Swiss, Moroccan, and Philippine delegations. However, ISDI goes by different names, some resembling pro-breastfeeding and infant health associations, but comprising top BMS companies. It is possible that not all associations were identified within the time constraints of this project.

## Conclusion

 The purpose of this study was to understand who participates in decision-making, and how actors frame and contest proposals to revise the Codex Standard on FUF. Subsequently, the results show that the revision procedures were dominated by HICs and industry groups, compared to limited middle- and low-income country, and civil society representation.

 The results also suggest that the baby food industry influences standard-setting processes at Codex, not only as observers, but also as part of member state delegations, which may raise political and public health concerns. Furthermore, industry stakeholders employ sophisticated strategies, such as diverting scientific research through sponsorship and cherry-picking results to use as evidence in proceedings, and at the same time continually arguing to narrow the scope for what can be considered scientific justification for regulation.

This study illustrates the need for Codex to minimize and mitigate conflicts-of-interest and consider the impact suboptimal standards can have on health. It also directs attention towards the importance of current and unbiased nutritional knowledge, through well-conducted high-quality research, to be reflected in Codex food standards to protect and benefit infants, children, mothers, and the wider global community. In addition, the results reiterate the important role *The Code*, WHA resolutions, and WHO guidance play in Codex BMS standards, through preserving breastfeeding, and regulating the marketing of BMS.

 Overall, this suggests that actions are needed to substantially increase support for LMIC, and civil society involvement at Codex. Such representation may help to counteract power asymmetries and commercial influences on food standards for infants and young children. The results may also remind member states that as voting participants at Codex, they are in a unique position to modify the food industry’s role in determining food standards for infants and young children.

## Acknowledgements

 PB, ML and DM thank the Department of Maternal, Newborn, Child and Adolescent Health, of the WHO for funding to support this study. ML is a member of the Food Standards Australia New Zealand (FSANZ) Board and the Australian National Health and Medical Research Council’s Synthesis and Translation of Research Evidence Committee. ML is responsible for the views expressed in this manuscript and they do not necessarily represent the views, decisions or policies of the institutions with which he is affiliated. The authors declare no other competing interests.

## Ethical issues

 No ethics approval was required as the study used secondary data only.

## Competing interests

 Authors declare that they have no competing interests.

## Authors’ contributions

 MB undertook this research project as part of a master’s thesis, including conception and design, acquisition of data, analysis and interpretation of data, and manuscript preparation. PB, ML, and CR were active supervisors of this manuscript. PB and ML were involved in the conception and design of this work. PB, ML, CR, KR, and DM provided analysis and interpretation of data, and critical revision of the manuscript.

## Disclaimer

 ML is a member of the FSANZ Board and the Australian National Health and Medical Research Council’s Synthesis and Translation of Research Evidence Committee. The author is responsible for the views expressed in this manuscript and they do not necessarily represent the views, decisions or policies of the institutions with which he is affiliated.

## Funding

 This research was supported by funding from the Department of Maternal, Newborn, Child and Adolescent Health, of the WHO. The study funder had no role in any stage of the research. The findings reported in this manuscript reflect the views and findings of the authors only, and do not necessarily represent those of the study funder.

## Supplementary files


Supplementary file 1. CCNFSDU Member State Delegates (2015-2017).

Supplementary file 2. CCNFSDU Member State Delegates (2018-2019).

Supplementary file 3. CCNFSDU Observers Categorized by Organization Type.

Supplementary file 4. Search Strategy.

Supplementary file 5. Comparison Between Codex and CCNFSDU Member State Representation by Country Income Level (2015-2019).
