# Supplementary material for: The Politics of Regulating Foods for Infants and Young Children: A Case Study on the Framing and Contestation of Codex Standard-Setting Processes on Breast-Milk Substitutes
Source: Int J Health Policy Manag. 2021 Nov 20;11(11):2422–39. doi: 10.34172/ijhpm.2021.161 (PMC9818087; doi:10.34172/ijhpm.2021.161)
Supplement: Supplementary file 1 — CCNFSDU Member State Delegates (2015-2017). [file ijhpm-11-2422-s001.pdf]

Article title: The Politics of Regulating Foods for Infants and Young Children: A Case Study on the Framing and Contestation of Codex Standard-Setting Processes on Breast-Milk Substitutes

Journal name: International Journal of Health Policy and Management (IJHPM)

Authors' information: Monique Boatwright[1]*, Mark Lawrence[2], Cherie Russell[1], Katheryn Russ[3], David McCoy[4], Phillip Baker[2]

[1]School of Exercise and Nutrition Sciences, Deakin University, Geelong, VIC, Australia.

[2]Institute for Physical Activity and Nutrition, School of Exercise and Nutrition, Deakin University, Geelong, VIC, Australia.

[3]University of California, Davis, CA, USA.

[4]Centre for Primary Care and Public Health, Queen Mary University, London, UK.

(*Corresponding author: mboatwright@deakin.edu.au)

Supplementary file 1. CCNFSDU Member State Delegates (2015-2017)

Table S1. Participants at CCNFSDU sessions (2015-2017)

List of participants at the 37th CCNFSDU session in Bad Soden am Taunus, Germany, 2015

Member states by income	low-income	lower-middle	upper-middle	high-income

Member Countries	Ministry of Agriculture	Ministry of Health	Ministry of Commerce	Other Ministry/Govt. Dept.	Dairy Industry	BMS Industry	Other Food Industry	Civil Society Groups	Academia	Other	Member State Delegation Totals
Algeria			1								1
Australia		1				1 Fonterra<br>1 Nestlé					3
Bangladesh						2 Nestlé		1 Bangladesh Breastfeeding Foundation			3
Belarus		1									1
Belgium		1									1
Brazil		2					1 Brazilian Association of Food Industries (ABIA)-Abbott		1 University of Sao Paulo	1 Brazilian Society of Pediatrics/UNIFESP	5
Cambodia			2								2

**Canada**		2					1 Neptune Technologies and Bioressources				3
**Chile**	1	1		1 Ministry of Exterior Relations							3
**China (People's Republic of)**		2		5 China National Center for Food Safety Risk Assesment<br>4 Entry-exit Inspection and Quarantine Bureau<br>1 Standard and Regulation Research Center<br>1 Centre for Food Safety, Food and EnvironmentalHygiene Department<br>1 Chinese Center for Disease Control and Prevention	1 Yili Industrial Group		2 China Food Information Center				17
**Colombia**							1 ANDI			1 INVIMA-Colombia National Food and Drug Surveillance Institute	2
**Costa Rica**		1									1
**Cuba**		1									1
**Côte D'Ivoire**							1 Fedération des Associations de Consommateurs Actifsde Côte d'Ivoire (FACACI)				1
**Denmark**	2						1 Danish Agriculture and Food Council				3
**Djibouti**			1								1
**Ecuador**		1									1
**Egypt**						1 Nestlé	1 Chamber of Food Industries			1 Egyptian Organization for Standardization and Quality (EOS)	3
**Equatorial Guinea**	2										2
**European Union**										1 Luxembourg Presidency<br>5 EU Commission<br>1 European Food Safety Authority (EFSA)<br>1 EU	8
**Finland**	1										1
**France**			1			3 Nutriset<br>1 Roquette					5
**Germany**	2					1 Nestlé	2 Diaetverbrand<br>1 BASF				9

Corresponding author: mboatwright@deakin.edu.au

							1 Merck Group<br>2 German Federation of Food Law and Food Science				
**Ghana**				2 Food & Drugs Authority<br>1 Standards Authority							3
**Hungary**				1 National Institute of Pharmacy & Nutrition							1
**India**		3									3
**Indonesia**				1 National Agency of Drug & Food Control<br>1 Ministry of Industry			1 DuPont<br>1 Southeast Asian Food & Agricultrure Science & Technology Center<br>2 APPNIA (Abbott, Fonterra, Frisian, Indofood, Mead Johnson, Nestlé, Kalbe Farma, Danone)<br>1 GAPMII (Indonesian Food & Beverage Asssoc.)				7
**Iran**		1									1
**Ireland**		1									1
**Italy**	2										2
**Japan**	1	1		2 Consumer Affairs Agency			1 National Institute of Health and Nutrition		1 Hokkaido University	1 International Life Sciences Institute Japan	7
**Kenya**				1 Kenya Bureau of Standards		1 Nestlé					2
**Kuwait**		1		1 Public Authority for Industry							2
**Latvia**	1										1
**Luxembourg**		1									1
**Malaysia**		2					1 Federation of Malaysian Manufacturers<br>1 Malaysian Palm Oil Board				4
**Mali**		1								1 Ambassador to Rome	2
**Mexico**				3 Federal Commission for the Protection against Sanitary Risk (COFEPRIS)	1 CANILEC					1 ILSI Mexico	5
**Morocco**	1					1 AMNI Moroccan Association for Child Nutrition (ISDI)				1 Official Laboratory of Chemical Analysis and Research<br>1 Moroccan Society of Pediatric Gastroenterology and Nutrition	4
**Nepal**	2										2
**Netherlands**		1									1

Corresponding author: mboatwright@deakin.edu.au

**New Zealand**	2				1 Fonterra<br>1 Dairy Goat Co-op<br>1 Nestlé						5
**Nigeria**	1										1
**Norway**	1	1				1 Nestlé					3
**Paraguay**		1									1
**Peru**		1									1
**Philippines**		2									2
**Poland**	1	1									2
**Republic of Korea**	1			4 Ministry of Food and Drug Safety							5
**Russian Federation**						1 Danone<br>1 Abbott	1 Coca-Cola<br>2 Russian Union of Industrialists and Entrepreneurs (RUIE)		1 Russian Institute of Nutrition		6
**Saudi Arabia**				1 Saudi Food and Drug Authority							1
**Senegal**									1 Université Cheikh Anta Diop de Dakar (UCAD)		1
**Singapore**	1										1
**Slovakia**		2									2
**South Africa**		3									3
**Spain**		1									1
**Sudan**				2 Sudanese Standards & Metrology Organisation							2
**Sweden**	1										1
**Switzerland**	2	1				1 Nestlé	2 Swiss Consumer Organizations				6
**Thailand**	2	2					4 Federation of Thai Industries				8
**Togo**	1										1
**Turkey**	1										1
**Uganda**		1		2 National Drug Authority							3
**United States of America**	2	1		3 US Food and Drug Administration		2 Infant Nutrition Council of America<br>1 Abbott					9
**Uruguay**		1									1

Corresponding author: mboatwright@deakin.edu.au

**Viet Nam**		1		1 Directorate for Standards & Quality							2
**Zimbabwe**		2									2
										**Total number of delegates**	**194**

List of participants at the 38th CCNFSDU session in Hamburg, Germany, 2016

Participating Member Countries	Ministry of Agriculture	Ministry of Health	Ministry of Commerce	Other Ministry/ Govt. Dept.	Dairy Industry	BMS Industry	Other Food Industry	Civil Society Groups	Academia	Other	Member State Delegation Totals
**Algeria**			1								1
**Australia**		1				1 Fonterra<br>1 Nestlé	1 Aspen Pharmacare				4
**Austria**		2									2
**Belgium**		1									1
**Brazil**		2					1 Brazilian Association of Food Industries (ABIA)		1 University of Sao Paulo	1 Brazilian Society of Paediatrics	5
**Canada**		3					1 DSM (DuPont)<br>1 Neptune Wellness Solution				5
**Chile**		1					1 DSM (DuPont)				2
**China (People's Republic of)**		5		3 China National Centre for Food Safety Risk Assessment<br>2 Centre for Food Safety, and Environmental Hygiene Department 1<br>Entry Exit Inspection and Quarantine Bureau<br>2 China Food and Drug Administration<br>1 China National Institute of Standardization			1 China Food Information Centre<br>1 China Nutrition and Health Food Association		2 National Institute for Nutrition and Health		18
**Colombia**							1 ANDI				1
**Costa Rica**		1									1
**Denmark**	1						1 Danish Agriculture and Food Council				2
**Ecuador**			1								1

Corresponding author: mboatwright@deakin.edu.au

**Egypt**						1 Hero<br>1 Nestlé	1 Chamber of Food Industries Egypt			1 EOS (Government Organization)<br>1 National Nutrition Institute (NNI)	5
**Estonia**	3										3
**European Union**										4 EU Commission<br>1 European Food Safety Authority (EFSA)	5
**Finland**	1										1
**France**			1			2 Nutriset	1 Alliance 7				4
**Germany**	4					1 Nestlé<br>1 Danone	1 BASF<br>1 Merck Group<br>2 German Federation for Food Law and Food Science (BLL)<br>2 Diaetverbrand				12
**Hungary**	1			1 National Institute of Pharmacy & Nutrition							2
**India**		3									3
**Indonesia**				2 National Agency of Drug & Food Control			1 APPNIA (Abbott, Fonterra, Frisian, Indofood, Mead Johnson, Nestlé, Kalbe Farma, Danone<br>2 GAPMII (Indonesian Food & Beverage Association)				5
**Ireland**		1									1
**Italy**	1										1
**Japan**	2	1					1 National Institute of Health and Nutrition		1 Hokkaido University		5
**Kenya**			1 Kenya Bureau of Standards			1 Nestlé					2
**Lesotho**				1 Food & Nutrition Coordination Office							1
**Malaysia**		2					1 Federation of Malaysian Manufacturers<br>3 Malaysian Palm Oil Council				6
**Mexico**		1		3 Federal Commission for the Protection against Sanitary Risk (COFEPRIS)	2 CANILEC	1 ANIPRON					7
**Morocco**	2					1 AMNI Moroccan Association	1 Département Recherche et Développement			1 Rabat Children's Hospital	6

Corresponding author: mboatwright@deakin.edu.au

						for Child Nutrition (ISDI)<br>1 Nestlé/AMNI	Société les Eaux Minérale d'Oulmes (Mineral Water)				
**Nepal**	3							1 Helen Keller International			4
**Netherlands**		1									1
**New Zealand**	2				1 Fonterra<br>1 Dairy Goat Co-op	1 Nestlé					5
**Nigeria**	1			2 Food Safety and Applied Nutrition<br>National Agency for Food and Drug<br>Administration and Control						2 Standards Organisation of Nigeria	5
**Norway**	1	1									2
**Panama**		1									1
**Paraguay**		1									1
**Peru**										1 Peru Consulate General	1
**Philippines**		1				1 Infant and Paediatric Nutrition Association of the Philippines (APIYCNA/ Abbott)					2
**Poland**	1								1 National Food and Nutrition Institute		2
**Republic of Korea**	1	3									4
**Russian Federation**				1 Federal Research Centre of Nutrition,<br>Biotechnology and Food Safety			2 Consumer Market Participants Union<br>2 Russian Union of Industrialists and Entrepreneurs (RUIE)				5
**Senegal**		1							1 Universite Gaston Berger<br>1 Université		3

 Corresponding author: mboatwright@deakin.edu.au

									Cheikh Anta Diop de Dakar (UCAD)		
**Singapore**		2									2
**Slovakia**		3								1 Permanent Representation of the Slovak Republic to the European Union<br>1 General Secretariat of the Council	5
**South Africa**		1							1 North-West University		2
**Sudan**				2 Sudanese Standards & Metrology							2
**Sweden**	1										1
**Switzerland**	2					1 Nestec S.A.	2 Swiss Consumer Organizations<br>1 DSM Nutritional Products Europe Ltd.,				6
**Thailand**	2	1					3 The Federation of Thai Industries				6
**Togo**	1										1
**Turkey**	1						1 Amway				2
**Uganda**				1 National Drug Authority						1 Presidential Assistant	2
**United Kingdom**		1									1
**United States of America**	3			4 U.S. Food and Drug Administration<br>2 USAID		1 Abbott<br>1 International Formula Council	1 Mars		1 University of Kansas Medical Center		13
**Uruguay**				1 Ministry of Foreign Affairs							1
**Viet Nam**		1		1 Directorate for Standards and Quality		1 Abbott					3
**Zimbabwe**		2									2
										**Total number of delegates**	**197**

List of participants at the 39th CCNFSDU session in Berlin, Germany, 2017

**Participating Member Countries**	**Ministry of Agriculture**	**Ministry of Health**	**Ministry of Commerce**	**Other Ministry/ Govt. Dept.**	**Dairy Industry**	**BMS Industry**	**Other Food Industry**	**Civil Society Groups**	**Academia**	**Other**	**Member State Delegation Totals**
**Algeria**			1								**1**

**Angola**				1 Ministry of National defence/Agriculture							**1**
**Argentina**	1										**1**
**Australia**		2			1 Fonterra	1 Nestlé	1 Aspen Pharmacare		1 University of Wollongong		**6**
**Austria**		2									**2**
**Belgium**		1									**1**
**Brazil**		2					1 Brazilian Association of Food Industries (ABIA)	1 IBFAN	1 Universidade Federal do Triangulo Mineiro<br>1 University of Sao Paulo		**6**
**Bulgaria**	1										**1**
**Burkina Faso**	1										**1**
**Cambodia**			1								**1**
**Canada**	1	2									**3**
**Chile**	1						1 DSM Nutritional Products (DuPont)				**2**
**China (People's Republic of)**				1 Department of Food Safety Standards<br>4 China National Center for Food Safety Risk Assessment<br>1 Centre for Food Safety, Food and Environmental Hygiene Department<br>2 Entry Exit Inspection and Quarantine Bureaux<br>2 Chinese<br>1 Center for Disease Control and Prevention<br>2 China Food and Drug Administration	1 Synlait		1 China Nutrion and Health Food Association				**15**
**Colombia**							1 Food and Nutrition Industry			1 INVIMA - Colombia National Food and Drug Surveillance Institute	**2**
**Costa Rica**		1	1								**2**
**Cuba**										1 Instituto de Higiene Epidemiología y Microbiología INHEM	**1**
**Denmark**	2						1 Danish Agriculture & Food Council				**3**
**Ecuador**		1									**1**

 Corresponding author: mboatwright@deakin.edu.au

**Egypt**						1 Hero<br>1 Nestlé				1 Egyptian Organization for Standardization and Quality<br>1 National Nutrition Institute (NNI)	**4**
**Estonia**	3									1 Council of the EU	**4**
**European Union**										6 EU Commission<br>1 European Food Safety Authority (EFSA)	**7**
**Finland**	1										**1**
**France**			1			2 Nutriset	1 Alliance 7				**4**
**Germany**	6					1 Nestlé<br>1 Dupont	1 BASF<br>1 Merck Group<br>2 Diaetverbrand<br>1 German Federation for Food Law and Food Science (manufacturers & lawyers / consultants)<br>1 Dupont Holdings				**14**
**Ghana**				2 Food & Drugs Authority							**2**
**Greece**										1 Embassy of Greece	**1**
**Hungary**				1 National Institute of Pharmacy & Nutrition							**1**
**India**		2		1 Consumer Affairs							**3**
**Indonesia**				1 National Agency of Drug & Food Control		1 APPNIA (Abbott, Fonterra, Frisian, Indofood, Mead Johnson, Nestlé, Kalbe Farma, Danone<br>2 GAPMII (Indonesian Food & Beverage Asssoc.)					**4**
**Ireland**		1									**1**
**Italy**	1										**1**

 Corresponding author: mboatwright@deakin.edu.au

**Japan**	1	1		1 Consumer Affairs Agency		1 National Institute of Health and Nutrition<br>1 Japan Health & Nutrition Food Association			1 Hokkaido University		**6**
**Kenya**				2 Kenya Bureau of Standards		1 Nestlé					**3**
**Kuwait**				1 Department of Education							**1**
**Lebanon**	1										**1**
**Lithuania**		1									**1**
**Malaysia**		2					1 Federation of Malaysian Manufacturers<br>1 Malaysia Palm Oil Board				**4**
**Mali**		2									**2**
**Mexico**		1			3 CANILEC	1 ANIPRON<br>1 Abbott<br>1 Mead Johnson					**7**
**Morocco**	2	1				1 AMNI Moroccan Association for Child Nutrition (ISDI)<br>1 Nestlé/AMNI	1 Département Recherche et Développement Société les Eaux Minérale d'Oulmes (mineral water)			1 Children's Hospital Rabat	**7**
**Nepal**	1									1 unknown	**2**
**Netherlands**		1									**1**
**New Zealand**	2				1 Fonterra<br>1 Dairy Goat Co-op						**4**
**Nigeria**	1			1 Food Safety and Applied Nutrition Directorate National Agency for Food and Drug Administration and Control (NAFDAC)						1 Standards Organisation of Nigeria	**3**
**Norway**		1		1 Norwegian Food Safety Authority							**2**

Corresponding author: mboatwright@deakin.edu.au

**Philippines**		1				1 Infant and Pediatric Nutrition Association of the Philippines (APIYCNA)/(Abbott)					**2**
**Poland**	1						1 Polish Federation of Food Industry Union of Employers		1 National Food and Nutrition Institute		**3**
**Qatar**				1 Standards and Metrology Department							**1**
**Republic of Korea**	1			2 Food and Drug Safety							**3**
**Russian Federation**				2 Federal Research Centre of Nutrition, Biotechnology and Food Safety			2 Consumer Market Participants Union				**4**
**Saudi Arabia**				1 Saudi Food and Drug Authority							**1**
**Senegal**		2							1 Université Cheikh Anta Diop de Dakar (UCAD)		**3**
**Singapore**	2										**2**
**Slovakia**		2									**2**
**South Africa**		1							1 North West University		**2**
**Sri Lanka**		1									**1**
**Sudan**				2 Sudanese Standard & Metrology							**2**
**Sweden**	1										**1**
**Switzerland**	1					1 Swiss Association of Nutrition Industries - SANI (ISDI)<br>1 Nestlé	2 DSM Nutritional Products Europe Ltd (DuPont)				**5**
**Thailand**	2	2					1 The Federation of Thai Industries				**5**
**Togo**	1										**1**
**Turkey**	1										**1**
**Uganda**				1 National Drug Authority			1 Reco Industries (maize, corn-soya etc.)				**2**
**United Kingdom**	2	1									**3**

 Corresponding author: mboatwright@deakin.edu.au

**United States of America**	4			4 U.S. Food and Drug Administration<br>2 USAID<br>1 Office of the U.S. Trade Representative	1 International Dairy Foods Association	1 Abbott<br>1 International Formula Council<br>1 Mead Johnson			1 University of Kansas Medical Center	1 Gage & Associates	**17**
**Viet Nam**	1	1			2 Vietnam Diary Association	2 Vinamilk<br>1 Eneright Nutrition<br>1 Abbott<br>1 Mead Johnson					**9**
**Zimbabwe**		2									**2**
										**Total number of delegates**	**213**

Corresponding author: mboatwright@deakin.edu.au
