# Supplementary material for: The Politics of Regulating Foods for Infants and Young Children: A Case Study on the Framing and Contestation of Codex Standard-Setting Processes on Breast-Milk Substitutes
Source: Int J Health Policy Manag. 2021 Nov 20;11(11):2422–39. doi: 10.34172/ijhpm.2021.161 (PMC9818087; doi:10.34172/ijhpm.2021.161)
Supplement: Supplementary file 2 — CCNFSDU Member State Delegates (2018-2019). [file ijhpm-11-2422-s002.pdf]

Article title: The Politics of Regulating Foods for Infants and Young Children: A Case Study on the Framing and Contestation of Codex Standard-Setting Processes on Breast-Milk Substitutes

Journal name: International Journal of Health Policy and Management (IJHPM)

Authors' information: Monique Boatwright[1]*, Mark Lawrence[2], Cherie Russell[1], Katheryn Russ[3], David McCoy[4], Phillip Baker[2]

[1]School of Exercise and Nutrition Sciences, Deakin University, Geelong, VIC, Australia.

[2]Institute for Physical Activity and Nutrition, School of Exercise and Nutrition, Deakin University, Geelong, VIC, Australia.

[3]University of California, Davis, CA, USA.

[4]Centre for Primary Care and Public Health, Queen Mary University, London, UK.

(*Corresponding author: mboatwright@deakin.edu.au)

Supplementary file 2. CCNFSDU Member State Delegates (2018-2019)

Table S2. Participants at CCNFSDU sessions (2018-2019)

List of participants at the 40th CCNFSDU session in Berlin, Germany, 2018

Member states by income	low-income	lower-middle	upper-middle	high-income

Member Countries	Ministry of Agriculture	Ministry of Health	Ministry of Commerce	Other Ministry/Govt. Dept.	Dairy Industry	BMS Industry	Other Food Industry	Civil Society Groups	Academia	Other	Member State Delegation Totals
**Argentina**	1	2			1 Centro de la Industria Lechera						**4**
**Australia**		2				1 Nestlé<br>1 Nutchev					**4**
**Austria**	1	2									**3**
**Azerbaijan**				2 Food Safety Agency							**2**
**Bangladesh**								1 Bangladesh Breastfeeding Foundation			**1**
**Belgium**		1									**1**

**Brazil**		2					1 Brazilian Association of Food Industries (ABIA)	1 IBFAN	1 University of Sao Paulo<br>1 Universidade Federal do Triangulo Mineiro		**6**
**Burkina Faso**	1										**1**
**Cambodia**			1					1 Helen Keller International			**2**
**Cameroon**		1		1 Ministry of Mines, Industry & Technological Development							**2**
**Canada**	1	3							1 University of Toronto		**5**
**Chile**	1	1					1 DSM Nutritional Product (DuPont)				**3**
**China (People's Republic of)**	4	1		6 China National Center for Food Safety Risk Assessment<br>2 State Administration for Market Regulation (SAMR)<br>1 Centre for Food Safety, Food and Environmental Hygiene Department			6 China Nutrition and Health Food Association<br>1 China National Food Industry Association				**21**
**Colombia**				1 Ministry of Exterior Relations					1 University el Bosque		**2**
**Costa Rica**		1	1							1 Private Sector	**3**
**Croatia**		1									**1**
**Cuba**		1									**1**
**Côte D'Ivoire**		2				1 Danone					**3**
**Denmark**	1						1 Danish Agriculture & Food Council				**2**
**Ecuador**		2									**2**
**Egypt**				1 Egyptian Organization for Standardization and Quality (EOS)		1 Hero<br>1 Nestlé	1 PepsiCo<br>1 Nestlé Waters			1 National Nutrition Institute (NNI)	**6**
**Estonia**	1										**1**
**European Union**	1			5 EU Commission							**6**
**Finland**	2										**2**
**France**			1	1 General Secretariate for European Affairs		2 Nutriset	1 Alliance 7<br>2 Servair				**7**
**Georgia**	1										**1**
**Germany**	3					1 Nestlé<br>1 Danone	1 Food-PharmaOTC<br>1 Diätverband<br>1 German Federation for Food Law and Food Science (manufacturers & lawyers/consultants)<br>1 DuPont Holdings		1	1 Maria Dubitsky Consulting (Food Law)	**11**

 Corresponding author: mboatwright@deakin.edu.au

**Ghana**				2 Food & Drugs Authority							**2**
**Greece**										1 Embassy of Greece in Berlin	**1**
**Guatemala**										1 Guatemala Embassy	**1**
**India**				1 Consumer Affairs 2 Food Safety & Standards Authority						1 ICMR - National Institute of Nutrition	**4**
**Indonesia**		1		2 National Agency of Drug & Food Control		1 APPNIA (Abbott, Fonterra, Frisian, Indofood, Mead Johnson, Nestlé, Kalbe Farma, Danone)<br>2 GAPMII (Indonesian Food & Beverage Asssoc.)				1 Embassy of the Rebublic of Indonesia	**7**
**Iran**										1 Private Sector	**1**
**Ireland**		2									**2**
**Italy**	2										**2**
**Jamaica**		1									**1**
**Japan**	1	1		1 Consumer Affairs Agency			2 National Institute of Health and Nutrition		1 Hokkaido University		**6**
**Kazakhstan**		3								1 Kazakh Academy of Nutrition	**4**
**Kenya**	2	1		1 Kenya Bureau of Standards<br>1 Regulations and Compliance		1 Nestlé Kenya Limited					**6**
**Kuwait**				1 Department of Education							**1**
**Lao People's Democratic Republic**		2						1 Save the Children			**3**
**Lithuania**		1									**1**
**Malaysia**		2				1 Mead Johnson	1 Malaysia Palm Oil Board				**4**
**Mali**		2									**2**
**Mexico**		3			1 CANILEC	1 Abbott<br>1 ANIPRON<br>1 Mead Johnson Nutrition					**7**

Corresponding author: mboatwright@deakin.edu.au

**Morocco**	2					1 AMNI Moroccan Association for Child Nutrition (ISDI)<br>1 Nestlé/AMNI				1 Children's Hospital Rabat	**5**
**Nepal**	1							1 Helen Keller International			**2**
**Netherlands**		1									**1**
**New Zealand**	2				1 Fonterra<br>1 Dairy Goat Co-op		1 Danisco-DuPont				**5**
**Niger**		1									**1**
**Nigeria**	1			1 Federal Ministry of Science and Technology<br>2 National Agency for Food and Drug Administration and Control (NAFDAC)			1 Association of Food, Beverage and Tobacco Employers (AFBTE)				**5**
**Norway**		1		1 Norwegian Food Safety Authority							**2**
**Paraguay**		1		1 National Institute of Technology, Standardization and Metrology (INTN)							**2**
**Peru**		1								1 Embassy to Germany	**2**
**Philippines**		2				1 Infant Nutrition Association of the Philippines (IPNAP) - (Abbott, Nestlé, Wyeth, Danone, Friesland/Campina, Reckitt Benckiser)					**3**
**Poland**	1										**1**
**Republic of Korea**	1			4 Ministry of food and drug safety							**5**
**Russian Federation**				2 Federal Research Centre of Nutrition, Biotechnology and Food Safety			1 Consumer Market Participants Union				**3**
**Saudi Arabia**				2 Saudi Food and Drug Authority							**2**
**Senegal**		1									**1**
**Singapore**	2										**2**
**Slovakia**		2									**2**

 Corresponding author: mboatwright@deakin.edu.au

**South Africa**		1							1 North West University		**2**
**Sri Lanka**		1							1 University of Colombo		**2**
**Sudan**							1 Dar Savanna Ltd (Gum Arabic)				**1**
**Sweden**	1										**1**
**Switzerland**	1					1 Swiss Association of Nutrition Industries - SANI<br>1 Nestlé	1 DSM Nutritional Products Europe Ltd				**4**
**Thailand**	2	1					2 The Federation of Thai Industries<br>1 Pediatric Nutrition Manufacturer Association of Thailand				**6**
**Uganda**				1 National Drug Authority			1 Reco Industries				**2**
**United Kingdom**	2	1		1 Food Standards Agency (FSA)							**4**
**United Republic of Tanzania**				1 Tanzania Bureau of Standards							**1**
**United States**	4			5 U.S. Food and Drug Administration<br>2 USAID	1 U.S. Dairy Export Council	1 Mead Johnson<br>1 Abbott<br>1 Infant Nutrition Council of America (INCA) - (Abbott, Gerber, Perrigo, Reckitt Benckiser)	1 Corn Refiners Association		1 University of Kansas Medical Center		**17**
**Uruguay**		1									**1**
**Viet Nam**		2			1 Vietnam Dairy Association	1 Vinamilk<br>1 Abbott<br>1 Reckitt Benckiser<br>2 Yakult					**8**
**Zimbabwe**		2									**2**
										**Total number of delegates**	**253**

List of participants at the 41st CCNFSDU session in Duesseldorf, Germany, 2019

**Participating Member Countries**	**Ministry of Agriculture**	**Ministry of Health**	**Ministry of Commerce**	**Other Ministry/ Govt. Dep.**	**Dairy Industry**	**BMS Industry**	**Other Food Industry**	**Civil Society Groups**	**Academia**	**Other**	**Member State**

Corresponding author: mboatwright@deakin.edu.au

											Delegation Totals
**Algeria**			1								**1**
**Argentina**	1	2			1 Técnico Centro de la Industria Lechera	2 Asociación de Empresas de Nutrición Infantil (ANI)					**6**
**Australia**		3				1 Nuchev Food					**4**
**Austria**		2									**2**
**Bangladesh**										1 Bangladesh Standards and Testing Institution	**1**
**Belgium**		1									**1**
**Botswana**	1	3								1 Ambassador to Germany	**5**
**Brazil**		2					1 ABIA – Brazilian Association of Food Industries<br>1 Confederação Nacional da Indústria - CNI		1 University of Sao Paulo		**5**
**Burkina Faso**	1	1									**2**
**Cambodia**			1					1 Helen Keller International			**2**
**Cameroon**	1			1 Mines, Industry and Technology							**2**
**Canada**	1	3									**4**
**Chile**		2									**2**
**China (People's Republic of)**	3			1 China National Center for Food Safety Risk Assessment			7 China Nutrition and Health Food Association		1 Institute of Food Science and Technology CAAS		**12**
**Colombia**		1									**1**
**Costa Rica**			1				1 Cámora Costarricense de la Industria Alimentario - CACIA				**2**
**Croatia**		1									**1**
**Cuba**		1									**1**
**Denmark**	1									1 unknown	**2**
**Ecuador**		1									**1**
**Egypt**				1 National Food Safety Authority		1 Nestlé<br>1 Hero<br>1 Riri Baby Food Co.	1 PepsiCo<br>1 Coca-Cola Atlantic Industries<br>1 Nestlé Waters		1 National Nutrition Institute (NNI)		**8**
**Estonia**	1										**1**
**European Union**										5 European Commission	**5**
**Finland**	2									1 Council of the European Union	**3**

 Corresponding author: mboatwright@deakin.edu.au

**France**			1	1 General Secretariate for European Affairs							**2**
**Gambia**										1 unkown	**1**
**Germany**	2			1 Federal Institute for Risk Assessment<br>1 Consumer Protection & Food Safety	1 Association of the German Dairy Industry	1 Nestlé<br>1 Nutricia Research (Danone)	1 BASF Nutrition<br>3 Federation of German Foods Industry<br>1 Diätverband e.V.<br>1 Proctor & Gamble Health			1 Maria Dubitsky Consulting (Food Law)	**14**
**Ghana**				2 Food & Drugs Authority							**2**
**Hungary**				1 National Institute of Pharmacy & Nutrition							**1**
**India**		2		1 Science & Technology<br>1 Consumer Affairs, Food & Public Distribution							**4**
**Indonesia**				1 National Agency of Drug & Food Control<br>2 Ministry of Industry		2 APPNIA (Abbott, Fonterra, Frisian, Indofood, Mead Johnson, Nestlé, Kalbe Farma, Danone)	2 The Indonesian Food and Beverages Association		1 Bogor Agricultural University		**8**
**Iran**										1 Private Sector	**1**
**Ireland**				2 Food Safety Authority							**2**
**Italy**	1										**1**
**Japan**		2		2 Consumer Affairs Agency			2 National Institute of Health and Nutrition				**6**
**Jordan**				1 Jordan Standard and Metrology Organization							**1**
**Kazakhstan**		2									**2**
**Kenya**	2					1 Nestlé					**3**
**Kuwait**		1									**1**
**Lao People's Democratic Republic**		4						1 Save the Children			**5**
**Lithuania**		1									**1**
**Malaysia**		2				1 Mead Johnson<br>2 Yakult	1 Malaysia Palm Oil Board			1 Nutrition Society of Malaysia (public/private)	**7**
**Mali**		1									**1**
**Morocco**	1	2		1 Department of pharmacy					1 Tofail University<br>1 Medicines and Pharmacy Directorate	1 Moroccan Society of Pediatric Gastroenterology and Nutrition	**7**
**Nepal**	1							1 Helen Keller International			**2**

 Corresponding author: mboatwright@deakin.edu.au

**Netherlands**		1									**1**
**New Zealand**	2				1 Fonterra<br>1 Dairy Goat Co-operative						**4**
**Niger**		1								1 Ambassador to Italy	**2**
**Nigeria**				1 National Agency for Food and Drug Administration and Control							**1**
**Norway**	1	1									**2**
**Panama**			1								**1**
**Paraguay**		1									**1**
**Peru**		2								1 ADEX, Exporters Association	**3**
**Philippines**		1		1 Food & Drug Administration		1 Infant Nutrition Association of the Philippines (IPNAP) - (Abbott, Nestlé, Wyeth, Danone, Friesland/Campina, Reckitt Benckiser)					**3**
**Poland**	1								1 National Food and Nutrition Institute		**2**
**Republic of Korea**				4 Food & Drug Safety							**4**
**Russian Federation**				2 Federal Research Centre of Nutrition, Biotechnology and Food Safety			3 Consumer Market Participants Union				**5**
**Saudi Arabia**				3 Saudi Food and Drug Authority							**3**
**Senegal**		1							1 Université Gaston Berger		**2**
**Singapore**	2										**2**
**Slovakia**		1									**1**
**South Africa**		1									**1**
**Spain**		1									**1**
**South Sudan**				1 South Sudan National Bureau of Standards			2 South Sudan Gum Arabic Federal Union		1 Juba University		**4**
**Spain**		1 Spanish Agency for Food Safety and Nutrition									**1**
**Sudan**				2 Sudanese Standards and Metrology Organisation							**2**

Corresponding author: mboatwright@deakin.edu.au

**Sweden**	1										**1**
**Switzerland**	1					1 Swiss Association of Nutrition Industries - SANI (ISDI)<br>1 Nestec S.A. (Nestlé)	1 DSM Nutrition Products (DuPont)				**4**
**Thailand**	1	1					3 Federation of Thai Industries		1 Mahidol University		**6**
**Turkey**	3						1 Association on Food Supplements and Nutrition				**4**
**United Kingdom**		1		1 Food Standards Agency<br>1 Global Affairs						1 unknown	**4**
**United Republic of Tanzania**				1 Bureau of Standards				1 World Alliance for Breastfeeding Action (WABA)			2
**United States of America**	4			4 U.S. Food and Drug Administration<br>2 USAID	1 U.S. Dairy Export Council	1 Mead Johnson<br>1 Infant Nutrition Council of America (INCA) - (Abbott, Gerber, Perrigo, Reckitt Benckiser)	1 Corn Refiners Association			1 U.S. Codex Office	**15**
**Viet Nam**		1			1 Vietnam Dairy Association<br>1 Mon Que Foodchain (TH Group)	7 Nawson Food & Beverage (Vinamilk)<br>1 Abbott<br>2 Yakult				1 Vietnam Codex Office<br>5 unknown<br>1 Vietnam Economics Association	**21**
**Zimbabwe**		1									**1**
										**Total number of delegates**	**250**

 Corresponding author: mboatwright@deakin.edu.au
