# Supplementary material for: The Politics of Regulating Foods for Infants and Young Children: A Case Study on the Framing and Contestation of Codex Standard-Setting Processes on Breast-Milk Substitutes
Source: Int J Health Policy Manag. 2021 Nov 20;11(11):2422–39. doi: 10.34172/ijhpm.2021.161 (PMC9818087; doi:10.34172/ijhpm.2021.161)
Supplement: Supplementary file 3 — CCNFSDU Observers Categorized by Organization Type. [file ijhpm-11-2422-s003.pdf]

**Article title:** The Politics of Regulating Foods for Infants and Young Children: A Case Study on the Framing and Contestation of Codex Standard-Setting Processes on Breast-Milk Substitutes

**Journal name:** International Journal of Health Policy and Management (IJHPM)

**Authors' information**: Monique Boatwright[1]*, Mark Lawrence[2], Cherie Russell[1], Katheryn Russ[3], David McCoy[4], Phillip Baker[2]

[1]School of Exercise and Nutrition Sciences, Deakin University, Geelong, VIC, Australia.

[2]Institute for Physical Activity and Nutrition, School of Exercise and Nutrition, Deakin University, Geelong, VIC, Australia.

[3]University of California, Davis, CA, USA.

[4]Centre for Primary Care and Public Health, Queen Mary University, London, UK.

(*Corresponding author: mboatwright@deakin.edu.au)

## **Supplementary file 3.** CCNFSDU Observers Categorized by Organization Type

**Table S3.** Observers at CCNFSDU meetings (2015-2019)

**Observers who attended the 37th Codex Committee on Nutrition and Foods for Special Dietary Uses (CCNSFDU) session in Bad Soden am Taunus, Germany, 2015**

| Codex category | Name of organization | Number of observers | Type of organization |
|---|---|---|---|
| IGO | Inter-American Institute for Cooperation on Agriculture (IICA) | 1 | IGO |
| NGO | Association internationale pour le développement des gommes naturelles (AIDGUM) | 1 | Industry |
| NGO | Association for International Promotion of Gums (AIPG) | 1 | Industry |
| NGO | AOAC International | 1 | Industry |
| NGO | Association of European Coeliac Societies (AOECS) | 1 | NGO |
| NGO | Calorie Control Council (CCC) | 2 | Industry |
| NGO | Comité européen des fabricants de sucre (CEFS) | 2 | Industry |
| NGO | Council for Responsible Nutrition (CRN) | 5 | Industry |
| NGO | Federation of European Specialty Food Ingredients Industries (ELC) | 4 | Industry |
| NGO | Early Nutrition Academy (ENA) | 1 | Industry |
| NGO | European Network of Childbirth Associations (ENCA) | 3 | NGO |
| NGO | European Society for Paediatric Gastroenterology Hepatology and Nutrition (ESPGHAN) | 1 | Industry |
| NGO | European Vegetable Protein Association (EUVEPRO) | 1 | Industry |
| NGO | FoodDrinkEurope | 4 | Industry |
| NGO | Global Organization for EPA and DHA Omega-3s (GOED) | 1 | Industry |
| NGO | Helen Keller International (HKI) | 1 | NGO |
| NGO | International Association of Consumer Food Organizations (IACFO) | 1 | Industry |
| NGO | International Alliance of Dietary/Food Supplement Associations (IADSA) | 9 | Industry |

| | | | |
|---|---|---|---|
| NGO | International Baby Food Action Network (IBFAN) | **3** | NGO |
| NGO | International Co-operative Alliance (ICA) | **2** | Industry |
| NGO | International Council on Amino Acid Science (ICAAS) | **1** | Industry |
| NGO | International Council of Beverages Associations (ICBA) | **2** | Industry |
| NGO | International Chewing Gum Association (ICGA) | **1** | Industry |
| NGO | International Council of Grocery Manufacturers Associations (ICGMA) | **2** | Industry |
| NGO | International Dairy Federation (IDF/FIL) | **5** | Industry |
| NGO | International Food Additives Council (IFAC) | **1** | Industry |
| NGO | Institute of Food Technologists (IFT) | **3** | 2 NGO (academia)/1 industry |
| NGO | International Glutamate Technical Committee (IGTC) | **1** | Industry |
| NGO | International Lactation Consultant Association (ILCA) | **1** | NGO |
| NGO | International Life Sciences Institute (ILSI) | **3** | 3 Industry |
| NGO | International Special Dietary Foods Industries (ISDI) | **12** | Industry |
| NGO | International Food Policy Research Institute (IFPRI) | **2** | Industry |
| NGO | National Health Federation (NHF) | **2** | NGO |
| NGO | Organisation des Fabricants de produits Cellulosiques Alimentaires (OFCA) | **1** | Industry |
| NGO | Specialised Nutrition Europe (SNE) | **9** | Industry |
| UN | United Nations Children's Fund (UNICEF) | **2** | IGO |
| N/A | Codex Secretariat | **5** | IGO |
| N/A | Food and Agriculture Organization (FAO) | **1** | IGO |
| N/A | World Health Organization (WHO) | **3** | IGO |
| N/A | CCNFSDU Host Secretariat | **2** | IGO |
| | **Total observers** | **104** | |

**Observers who attended the 38th Codex Committee on Nutrition and Foods for Special Dietary Uses (CCNSFDU) session in Hamburg, Germany, 2016**

| Codex category | Name of organization | Number of observers | Type of organization |
|---|---|---|---|
| Special Observer | Palestine Standards Institution | **1** | Govt. |
| Special Observer | Palestine Ministry of Health | **1** | Govt. |
| Special Observer | Standards and Trade Secretariat (African Union) | **1** | Govt. |
| Special Observer | Food Safety Officer (African Union) | **1** | Govt. |
| NGO | Association européenne pour le droit de l'alimentation (AEDA/EFLA) | **1** | NGO |
| IGO | Inter-American Institute for Cooperation on Agriculture (IICA) | **1** | IGO |
| NGO | AOAC International | **2** | Industry |
| NGO | American Oil Chemist's Society (AOCS) | **1** | Industry |
| NGO | Association of European Coeliac Societies (AOECS) | **1** | NGO |
| NGO | Calorie Control Council (CCC) | **2** | Industry |
| NGO | Conseil européen de l'industrie chimique (CEFIC) | **1** | Industry |
| NGO | Council for Responsible Nutrition (CRN) | **3** | Industry |
| NGO | Federation of European Specialty Food Ingredients Industries (ELC) | **5** | Industry |
| NGO | European Network of Childbirth Associations (ENCA) | **1** | NGO |
| NGO | European Society for Paediatric Gastroenterology Hepatology and Nutrition (ESPGHAN) | **2** | Industry |
| NGO | European Vegetable Protein Association (EUVEPRO) | **3** | Industry |
| NGO | Food Industry Asia (FIA) | **1** | Industry |
| NGO | FoodDrinkEurope | **5** | Industry |
| NGO | Global Organization for EPA and DHA Omega-3s (GOED) | **1** | Industry |
| NGO | Helen Keller International (HKI) | **3** | NGO |


Corresponding author: mboatwright@deakin.edu.au

| | | | |
|---|---|---|---|
| NGO | International Association of Consumer Food Organizations (IACFO) | **1** | Industry |
| NGO | International Alliance of Dietary/Food Supplement Associations (IADSA) | **7** | Industry |
| NGO | International Baby Food Action Network (IBFAN) | **2** | NGO |
| NGO | International Co-operative Alliance (ICA) | **2** | Industry |
| NGO | International Council on Amino Acid Science (ICAAS) | **2** | Industry |
| NGO | International Council of Beverages Associations (ICBA) | **3** | Industry |
| NGO | International Chewing Gum Association (ICGA) | **1** | Industry |
| NGO | International Council of Grocery Manufacturers Associations (ICGMA) | **2** | Industry |
| NGO | International Dairy Federation (ADF/FIL) | **4** | Industry |
| NGO | International Food Additives Council (IFAC) | **2** | Industry |
| NGO | Institute of Food Technologists (IFT) | **2** | NGO (academia) |
| NGO | International Lactation Consultant Association (ILCA) | **1** | NGO |
| NGO | International Life Sciences Institute (ILSI) | **4** | 3 Industry/1 NGO (academia) |
| NGO | International Probiotics Association (IPA) | **1** | Industry |
| NGO | International Special Dietary Foods Industries (ISDI) | **10** | Industry |
| NGO | International Food Policy Research Institute (IFPRI) | **3** | Industry |
| NGO | Médecins Sans Frontières International (MSF) | **2** | NGO |
| NGO | National Health Federation (NHF) | **3** | NGO |
| NGO | Specialised Nutrition Europe (SNE) | **9** | Industry |
| NGO | Association of Yoghurts & Live fermented milks (YLFA) | **1** | Industry |
| UN | United Nations Children's Fund (UNICEF) | **1** | IGO |
| N/A | Codex Secretariat | **3** | IGO |
| N/A | Food and Agriculture Organization (FAO) | **3** | IGO |
| N/A | World Health Organization (WHO) | **3** | IGO |
| N/A | CCNFSDU Host Secretariat | **3** | IGO |
| | **Total observers** | **112** | |

**Observers who attended the 39th Codex Committee on Nutrition and Foods for Special Dietary Uses (CCNSFDU) session in Berlin, Germany, 2017**

| **Codex category** | **Name of organization** | **Number of observers** | **Type of organization** |
|---|---|---|---|
| Special Observer | Technical Consulatant (Palestine) | **1** | Govt. |
| Special Observer | Palestine Ministry of Health | **1** | Govt. |
| Special Observer | Standards and Trade Secretariat, Interafrican Bureau for Animal Resources (African Union) | **1** | Govt. |
| NGO | Association européenne pour le droit de l'alimentation (AEDA/EFLA) | **1** | NGO |
| NGO | AOAC International | **2** | Industry |
| NGO | American Oil Chemist's Society (AOCS) | **1** | Industry |
| NGO | Association of European Coeliac Societies (AOECS) | **1** | NGO |
| NGO | Calorie Control Council (CCC) | **1** | Industry |
| NGO | Council for Responsible Nutrition (CRN) | **5** | Industry |
| NGO | Early Nutrition Academy (ENA) | **1** | Industry |
| NGO | European Society for Paediatric Gastroenterology Hepatology and Nutrition (ESPGHAN) | **1** | NGO |
| NGO | Federation of European Specialty Food Ingredients Industries | **5** | Industry |
| NGO | European Vegetable Protein Association (EUVEPRO) | **1** | Industry |
| NGO | FoodDrinkEurope | **3** | Industry |
| NGO | Global Organization for EPA and DHA Omega-3s (GOED) | **5** | Industry |
| NGO | Helen Keller International (HKI) | **4** | NGO |
| NGO | International Association of Consumer Food Organizations (IACFO) | **1** | Industry |
| NGO | International Alliance of Dietary/Food Supplement Associations (IADSA) | **4** | Industry |
| NGO | International Baby Food Action Network (IBFAN) | **2** | NGO |
| NGO | International Co-operative Alliance (ICA) | **2** | Industry |

 Corresponding author: mboatwright@deakin.edu.au

| | | | |
|---|---|---|---|
| NGO | International Council on Amino Acid Science (ICAAS) | **4** | Industry |
| NGO | International Council of Beverages Associations (ICBA) | **3** | Industry |
| NGO | International Chewing Gum Association (ICGA) | **1** | Industry |
| NGO | International Council of Grocery Manufacturers Associations (ICGMA) | **1** | Industry |
| NGO | International Dairy Federation (ADF/FIL) | **4** | Industry |
| NGO | International Federation of Margarine Associations (IFMA) | **1** | Industry |
| NGO | Institute of Food Technologists (IFT) | **1** | NGO (academia) |
| NGO | International Fruit and Vegetable Juice Association | **1** | Industry |
| IGO | Inter-American Institute for Cooperation on Agriculture (IICA) | **1** | IGO |
| NGO | International Lactation Consultant Association (ILCA) | **1** | NGO |
| NGO | International Life Sciences Institute (ILSI) | **4** | 3 Industry/1 NGO (academia) |
| NGO | International Probiotics Association (IPA) | **6** | Industry |
| NGO | International Special Dietary Foods Industries (ISDI) | **12** | Industry |
| NGO | International Food Policy Research Institute (IFPRI) | **2** | Industry |
| NGO | Médecins Sans Frontières International (MSF) | **1** | NGO |
| NGO | National Health Federation (NHF) | **2** | NGO |
| NGO | Specialised Nutrition Europe (SNE) | **6** | Industry |
| NGO | United States Pharmacopeial Convention (USP) | **1** | Industry |
| UN | United Nations Children's Fund (UNICEF) | **1** | IGO |
| N/A | Codex Secretariat | **4** | IGO |
| N/A | Food and Agriculture Organization (FAO) | **3** | IGO |
| N/A | World Health Organization (WHO) | **6** | IGO |
| N/A | CCNFSDU Host Secretariat | **2** | IGO |
| | **Total observers** | **111** | |

**Observers who attended the 40th Codex Committee on Nutrition and Foods for Special Dietary Uses (CCNSFDU) session in Berlin, Germany, 2018**

| Codex category | Name of organization | Number of observers | Type of organization |
|---|---|---|---|
| Special Observer | Palestine Standards Institution | **1** | Govt. |
| Special Observer | Palestine Ministry of Health | **1** | Govt. |
| NGO | Action Contre La Faim (ACF) | **1** | NGO |
| NGO | Association européenne pour le droit de l'alimentation (AEDA) | **1** | NGO |
| NGO | Association for International Promotion of Gums (AIPG) | **1** | Industry |
| NGO | AOAC International | **2** | Industry |
| NGO | Association of European Coeliac Societies (AOECS) | **1** | NGO |
| NGO | American Society for Nutrition (ASN) | **1** | Industry |
| NGO | Calorie Control Council (CCC) | **1** | Industry |
| NGO | Comité européen des fabricants de sucre (CEFS) | **1** | Industry |
| NGO | Council for Responsible Nutrition (CRN) | **4** | Industry |
| NGO | Early Nutrition Academy (ENA) | **1** | Industry |
| NGO | European Network of Childbirth Associations (ENCA) | **1** | NGO |
| NGO | Federation of European Specialty Food Ingredients Industries | **6** | Industry |
| NGO | European Vegetable Protein Association (EUVEPRO) | **1** | Industry |
| NGO | Food Industry Asia (FIA) | **1** | Industry |
| NGO | FoodDrinkEurope | **7** | Industry |
| NGO | Global Organization for EPA and DHA Omega-3s (GOED) | **5** | Industry |
| NGO | Helen Keller International (HKI) | **6** | NGO |
| NGO | International Association of Consumer Food Organizations (IACFO) | **1** | Industry |

| | | | |
|---|---|---|---|
| NGO | International Alliance of Dietary/Food Supplement Associations (IADSA) | **7** | Industry |
| NGO | International Baby Food Action Network (IBFAN) | **1** | NGO |
| NGO | International Co-operative Alliance (ICA) | **1** | Industry |
| NGO | International Council on Amino Acid Science (ICAAS) | **4** | Industry |
| NGO | International Council of Beverages Associations (ICBA) | **6** | Industry |
| NGO | International Chewing Gum Association (ICGA) | **1** | Industry |
| NGO | International Dairy Federation (ADF/FIL) | **4** | Industry |
| NGO | International Food Additives Council (IFAC) | **2** | Industry |
| NGO | Institute of Food Technologists (IFT) | **2** | NGO (academia) |
| NGO | International Fruit and Vegetable Juice Association | **1** | Industry |
| IGO | Inter-American Institute for Cooperation on Agriculture (IICA) | **1** | IGO |
| NGO | International Lactation Consultant Association (ILCA) | **1** | NGO |
| NGO | International Life Sciences Institute (ILSI) | **4** | Industry |
| NGO | International Probiotics Association (IPA) | **10** | Industry |
| NGO | International Special Dietary Foods Industries (ISDI) | **21** | 20 Industry 1 Govt. |
| NGO | International Food Policy Research Institute (IFPRI) | **3** | 1 Industry 1 NGO |
| NGO | Médecins Sans Frontières International (MSF) | **2** | NGO |
| NGO | National Health Federation (NHF) | **2** | NGO |
| NGO | Specialised Nutrition Europe (SNE) | **5** | Industry |
| NGO | World Public Health Nutrition Association (WPHNA) | **3** | NGO |
| UN | United Nations Children's Fund (UNICEF) | **1** | IGO |
| N/A | Codex Secretariat | **4** | IGO |
| N/A | Food and Agriculture Organization (FAO) | **3** | IGO |
| N/A | World Health Organization (WHO) | **6** | IGO |
| N/A | CCNFSDU Host Secretariat | **4** | IGO |
| | **Total observers** | **143** | |

**Observers who attended the 41st Codex Committee on Nutrition and Foods for Special Dietary Uses (CCNSFDU) session in Duesseldorf, Germany, 2019**

| Codex category | Name of organization | Number of observers | Type of organization |
|---|---|---|---|
| Special Observer | Palestine Standards Institution | **1** | Govt. |
| IGO | Inter-American Institute for Cooperation on Agriculture (IICA) | **1** | IGO |
| NGO | Association internationale pour le développement des gommes naturelles (AIDGUM) | **1** | Industry |
| NGO | Association for International Promotion of Gums (AIPG) | **1** | Industry |
| NGO | Association of Manufacturers and Formulators of Enzyme Products (AMFEP) | **1** | Industry |
| NGO | AOAC International | **3** | Industry |
| NGO | American Oil Chemist's Society (AOCS) | **1** | Industry |
| NGO | Association of European Coeliac Societies (AOECS) | **1** | NGO |
| NGO | Council for Responsible Nutrition (CRN) | **3** | Industry |
| NGO | European Network of Childbirth Associations (ENCA) | **1** | NGO |
| NGO | Federation of European Specialty Food Ingredients Industries | **5** | Industry |
| NGO | European Vegetable Protein Association (EUVEPRO) | **4** | Industry |
| NGO | FoodDrinkEurope | **6** | Industry |
| NGO | Helen Keller International (HKI) | **4** | NGO |
| NGO | International Association for Cereal Science and Technology (IACST) | **2** | Industry |
| NGO | International Alliance of Dietary/Food Supplement Associations (IADSA) | **6** | Industry |
| NGO | International Baby Food Action Network (IBFAN) | **1** | NGO |
| NGO | International Council on Amino Acid Science (ICAAS) | **3** | Industry |
| NGO | International Council of Beverages Associations (ICBA) | **4** | Industry |
| NGO | International Chewing Gum Association (ICGA) | **2** | Industry |


Corresponding author: mboatwright@deakin.edu.au

| | | | |
|---|---|---|---|
| NGO | International Council of Grocery Manufacturers Associations (ICGMA) | **1** | Industry |
| NGO | International Dairy Federation (ADF/FIL) | **6** | Industry |
| NGO | International Food Additives Council (IFAC) | **1** | Industry |
| NGO | Institute of Food Technologists (IFT) | **1** | NGO (academia) |
| NGO | International Fruit and Vegetable Juice Association | **1** | Industry |
| NGO | International Lactation Consultant Association (ILCA) | **1** | NGO |
| NGO | International Life Sciences Institute (ILSI) | **3** | Industry |
| NGO | International Probiotics Association (IPA) | **5** | Industry |
| NGO | International Special Dietary Foods Industries (ISDI) | **23** | Industry |
| NGO | International Food Policy Research Institute (IFPRI) | **2** | 1 Industry 1 NGO |
| NGO | International Ready To Use Foods Association (IRUFA) | **3** | Industry |
| NGO | Médecins Sans Frontières International (MSF) | **3** | NGO |
| NGO | National Health Federation (NHF) | **1** | NGO |
| NGO | Specialised Nutrition Europe (SNE) | **6** | Industry |
| NGO | World Obesity Federation (WOF) | **1** | NGO |
| NGO | World Public Health Nutrition Association (WPHNA) | **1** | NGO |
| NGO | Association of Yoghurts & Live fermented milks (YLFA) | **3** | Industry |
| UN | United Nations Children's Fund (UNICEF) | **2** | IGO |
| N/A | Codex Secretariat | **5** | IGO |
| N/A | Food and Agriculture Organization (FAO) | **1** | IGO |
| N/A | World Health Organization (WHO) | **6** | IGO |
| N/A | CCNFSDU Host Secretariat | **3** | IGO |
| | **Total observers** | **130** | |

 Corresponding author: mboatwright@deakin.edu.au
