# Supplementary material for: The Politics of Regulating Foods for Infants and Young Children: A Case Study on the Framing and Contestation of Codex Standard-Setting Processes on Breast-Milk Substitutes
Source: Int J Health Policy Manag. 2021 Nov 20;11(11):2422–39. doi: 10.34172/ijhpm.2021.161 (PMC9818087; doi:10.34172/ijhpm.2021.161)
Supplement: Supplementary file 4 — Search Strategy. [file ijhpm-11-2422-s004.pdf]

Article title: The Politics of Regulating Foods for Infants and Young Children: A Case Study on the Framing and Contestation of Codex Standard-Setting Processes on Breast-Milk Substitutes

Journal name: International Journal of Health Policy and Management (IJHPM)

Authors' information: Monique Boatwright[1]*, Mark Lawrence[2], Cherie Russell[1], Katheryn Russ[3], David McCoy[4], Phillip Baker[2]

[1]School of Exercise and Nutrition Sciences, Deakin University, Geelong, VIC, Australia.

[2]Institute for Physical Activity and Nutrition, School of Exercise and Nutrition, Deakin University, Geelong, VIC, Australia.

[3]University of California, Davis, CA, USA.

[4]Centre for Primary Care and Public Health, Queen Mary University, London, UK.

(*Corresponding author: mboatwright@deakin.edu.au)

Supplementary file 4. Search Strategy

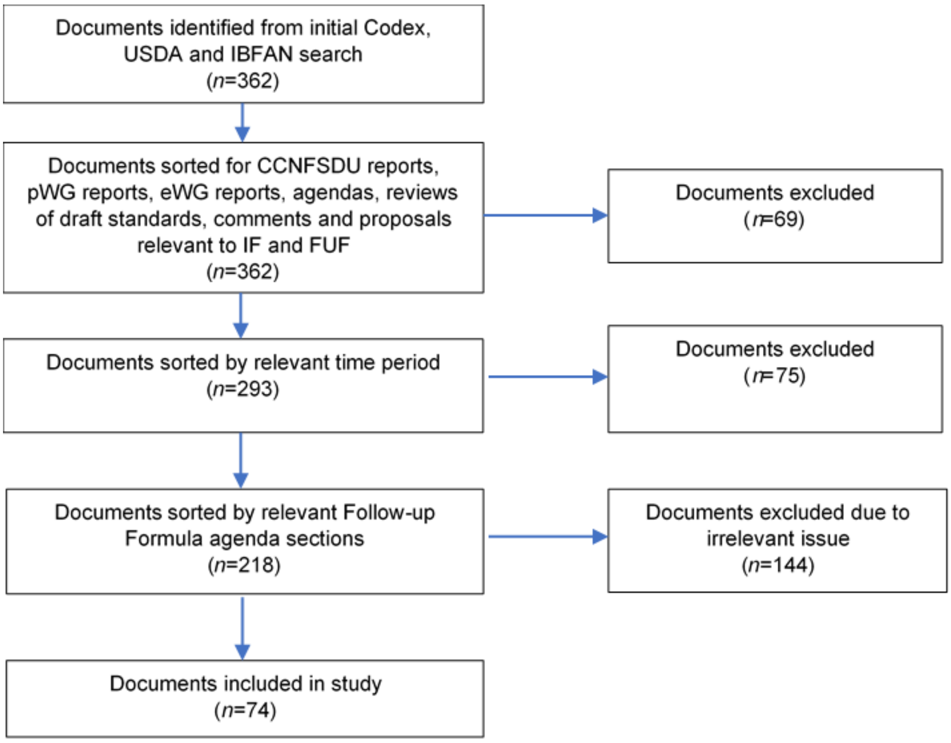

Figure S1. PRISMA summary of the search process for documents relating to deliberations on the Standard on Follow-up Formula at Codex Committee on Nutrition and Foods (CCNFSDU) meetings

Table S4. Inclusion and exclusion criteria applied in the document search process

**Inclusion Criteria**	**Exclusion Criteria**
Codex CNFSDU session reports, electronic working group reports, and expert meeting reports	Documents outside of 2015-2019
Codex CNFSDU background documents and discussion papers	Documents not in English
Reviews of the standard and proposed drafts for Follow-up Formula	Texts relating to the standard for Infant Formula, Formula for Special Medical Purposes, and Ready-to-use Therapeutic Foods
Codex CNFSDU comments of member states, delegates and observers relevant to additional labelling requirements, protein content and carbohydrates provisions	Reports/comments on aspects other than those specified e.g., biofortification, probiotics and trans fatty acids
