# Supplementary material for: The Politics of Regulating Foods for Infants and Young Children: A Case Study on the Framing and Contestation of Codex Standard-Setting Processes on Breast-Milk Substitutes
Source: Int J Health Policy Manag. 2021 Nov 20;11(11):2422–39. doi: 10.34172/ijhpm.2021.161 (PMC9818087; doi:10.34172/ijhpm.2021.161)
Supplement: Supplementary file 5 — Comparison Between Codex and CCNFSDU Member State Representation by Country Income Level (2015-2019). [file ijhpm-11-2422-s005.pdf]

Article title: The Politics of Regulating Foods for Infants and Young Children: A Case Study on the Framing and Contestation of Codex Standard-Setting Processes on Breast-Milk Substitutes

Journal name: International Journal of Health Policy and Management (IJHPM)

Authors' information: Monique Boatwright[1]*, Mark Lawrence[2], Cherie Russell[1], Katheryn Russ[3], David McCoy[4], Phillip Baker[2]

[1]School of Exercise and Nutrition Sciences, Deakin University, Geelong, VIC, Australia.

[2]Institute for Physical Activity and Nutrition, School of Exercise and Nutrition, Deakin University, Geelong, VIC, Australia.

[3]University of California, Davis, CA, USA.

[4]Centre for Primary Care and Public Health, Queen Mary University, London, UK.

(*Corresponding author: mboatwright@deakin.edu.au)

Supplementary file 5. Comparison Between Codex and CCNFSDU Member State Representation by Country Income Level (2015-2019)

Figure S2 compares the mean percentage of member state representation at Codex Alimentarius Commission (Codex) meetings and Codex Committee on Nutrition and Foods for Special Dietary Uses (CCNFSDU) meetings from 2015-2019, according to country income level. The results show that low-income countries are under-represented at the CCNFSDU relative to Codex as a whole, and that high-income countries are over-represented at the CCNFSDU relative to Codex as a whole.

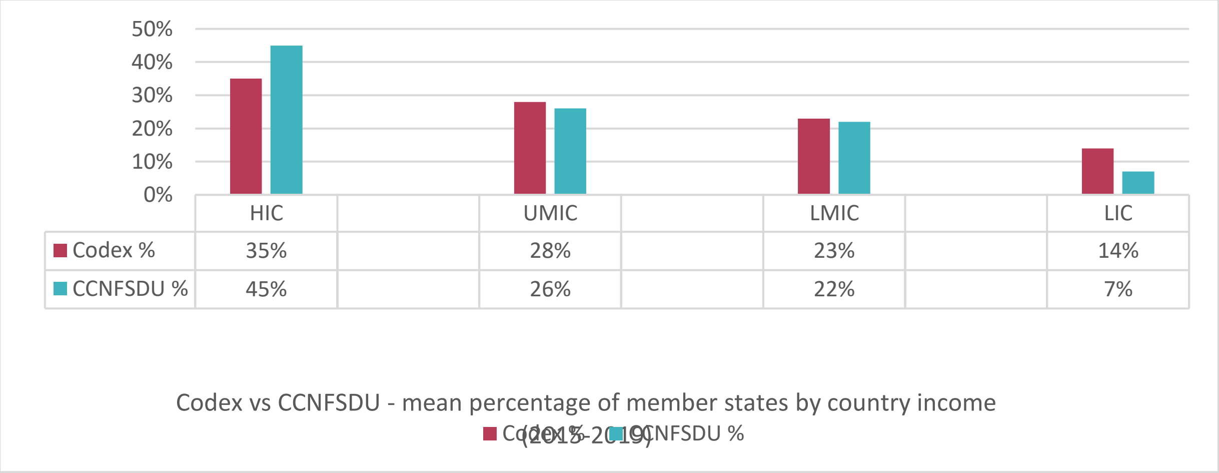

Figure S2. Mean percentage of member state delegations at Codex and CCNFSDU meetings based on country income (2015-2019). Abbreviations: CCNFSDU, Codex Committee on Nutrition and Foods for Special Dietary Uses; Codex, Codex Alimentarius Commission; HIC, high-income countries; UMIC, upper-middle-income countries; LMIC, lower-middle-income countries; LIC, low-income countries.
